# Deep learning-based image analysis in muscle histopathology using photo-realistic synthetic data

**DOI:** 10.1038/s43856-025-00777-y

**Published:** 2025-03-06

**Authors:** Leonid Mill, Oliver Aust, Jochen A. Ackermann, Philipp Burger, Monica Pascual, Katrin Palumbo-Zerr, Gerhard Krönke, Stefan Uderhardt, Georg Schett, Christoph S. Clemen, Christian Holtzhausen, Samir Jabari, Rolf Schröder, Andreas Maier, Anika Grüneboom

**Affiliations:** 1 MIRA Vision Microscopy GmbH, 73037 Göppingen, Germany; 2 Pattern Recognition Lab, Friedrich-Alexander University Erlangen-Nürnberg (FAU), 91058 Erlangen, Germany; 3 Department of Medicine 3 - Rheumatology and Immunology & Deutsches Zentrum für Immuntherapie, Friedrich-Alexander University Erlangen-Nürnberg (FAU) and Universitätsklinikum Erlangen, 91054 Erlangen, Germany; 4 Institute of Aerospace Medicine, German Aerospace Center (DLR), Cologne, Germany; 5 Institute of Vegetative Physiology, Medical Faculty, University of Cologne, Cologne, Germany; 6 Department of Neuropathology, Universitätsklinikum Erlangen, Friedrich-Alexander University Erlangen-Nürnberg (FAU), 91054 Erlangen, Germany; 7 Klinikum Nuremberg, Institute of Pathology, Paracelsus Medical University, 90419, Nuremberg, Germany; 8 Leibniz-Institut für Analytische Wissenschaften - ISAS - e.V, 44139 Dortmund, Germany

**Keywords:** Computational biology and bioinformatics, Medical research

## Abstract

**Background:**

Artificial intelligence (AI), specifically Deep learning (DL), has revolutionized biomedical image analysis, but its efficacy is limited by the need for representative, high-quality large datasets with manual annotations. While latest research on synthetic data using AI-based generative models has shown promising results to tackle this problem, several challenges such as lack of interpretability and need for vast amounts of real data remain. This study aims to introduce a new approach—SYNTA—for the generation of photo-realistic synthetic biomedical image data to address the challenges associated with state-of-the art generative models and DL-based image analysis.

**Methods:**

The SYNTA method employs a fully parametric approach to create photo-realistic synthetic training datasets tailored to specific biomedical tasks. Its applicability is tested in the context of muscle histopathology and skeletal muscle analysis. This new approach is evaluated for two real-world datasets to validate its applicability to solve complex image analysis tasks on real data.

**Results:**

Here we show that SYNTA enables expert-level segmentation of unseen real-world biomedical data using only synthetic training data. By addressing the lack of representative and high-quality real-world training data, SYNTA achieves robust performance in muscle histopathology image analysis, offering a scalable, controllable and interpretable alternative to generative models such as Generative Adversarial Networks (GANs) or Diffusion Models.

**Conclusions:**

SYNTA demonstrates great potential to accelerate and improve biomedical image analysis. Its ability to generate high-quality photo-realistic synthetic data reduces reliance on extensive collection of data and manual annotations, paving the way for advancements in histopathology and medical research.

## Plain language summary

Image analysis is essential for diagnosing diseases and advancing medical research. However, analyzing these images is challenging due to their complexity and the amount of data they contain. Artificial intelligence (AI) can help to improve this process but developing AI solutions requires large, high-quality training data which can be time-consuming and expensive to obtain. We developed an alternative computational approach called SYNTA to create realistic computer-generated images that can be used as training data for AI systems. We trained an AI system on computer-generated data only and show it can analyze previously unseen real biomedical images as accurately as human experts. This approach could make biomedical research easier, hence speeding up the process of developing new methods to detect disease from images.

## Abstract (web summary)

Mill et al. introduce SYNTA, a parametric method for generating synthetic, photorealistic biomedical images to train deep learning systems that does not use artificial intelligence. SYNTA enables expert-level segmentation on real-world data while overcoming domain shifts and offering a controllable alternative to generative models such as Generative Adversarial Networks and Diffusion Models.

## Introduction

Technical developments in the field of diagnostic and high throughput imaging are accompanied by an increasing generation of large-scale and complex image datasets^1–7^, which often rely on accurate and efficient quantitative data analysis^8–15^. The demands for precise diagnostics, the elucidation of disease mechanisms, and the identification of digital biomarkers have further intensified the need for robust and scalable image analysis techniques^16–19^. However, as manual or semi-automated analysis of such large datasets within an acceptable time frame has become increasingly difficult, there is a compelling need to transition from traditional methods to advanced approaches^20,21^.

Automated image analysis techniques are essential for the examination of histopathological images, as they offer rapid, objective, and reproducible assessments^8,9,11,17^. Traditional manual quantification of histopathological specimens is labor-intensive, prone to variability, and influenced by observer bias^22^. In contrast, automated approaches, especially those leveraging deep learning, provide consistent analysis of complex tissue structures, detection of subtle patterns, and efficient processing of large datasets. This results in a marked enhancement of accuracy and operational throughput. Consequently, these methods are pivotal in addressing the increasing demand for precise and high-throughput pathological assessment in both clinical and research environments.

In recent years, a variety of tools have been developed for the quantification of muscle tissue in histopathology images, encompassing manual^23^, semi-automated^24–28^, and fully automated AI-based approaches^29–33^. Manual techniques, while useful, are time-consuming and inherently subjective, rendering them suitable primarily for hand-selected small regions of interest (ROIs). These ROIs, however, often fail to represent the fiber morphology of the entire tissue section^32^. This limitation is particularly pronounced when analyzing whole slide images (WSIs) of muscle sections, which can contain tens of thousands of fibers^34^. In such cases, automated methods become indispensable to achieve comprehensive and accurate quantification.

Significant advancements in artificial intelligence, particularly in deep learning, have led to the development of several methods for automated quantification of images in muscle histopathology^15,32,34–41^. These AI-driven solutions demonstrate considerable potential to enhance the quality, objectivity, reproducibility, and speed of muscle slice analysis. However, current state-of-the-art deep learning approaches predominantly adhere to the supervised learning paradigm^42,43^, necessitating a substantial number of high-quality training images accompanied by labor-intensive manual annotations^22^. Moreover, these deep learning models are typically tailored to specialized datasets and often exhibit poor generalization to similar but distinct data, a phenomenon known as domain shift^44^. This shift can be attributed to variations of the tissue and staining protocols, sample preparation protocols, or even data acquisition hardware^44,45^. Consequently, the size and quality of the dataset represent a significant bottleneck, making the acquisition of representative datasets one of the most crucial factors in developing reliable deep learning models for muscle pathology^32^.

In recent years, various approaches have been proposed to address this problem, ranging from using machine learning techniques^46–54^, various labeling and annotation methods^50,55–57^ or to the collection of even bigger datasets^1–3,5,32,58^. A more recent and popular trend is the generation of synthetic photo-realistic images using AI-based generative models, such as Generative Adversarial Networks (GANs) and Diffusion Models (DMs)^59–69^. The primary goal is to artificially augment the size and quality of existing training datasets^59,70^. Generative models have diverse applications in biomedical image analysis, including image enhancement^71^, domain adaptation^59,63^, and the generation of new image samples based on existing datasets^70,72–74^. Several studies have demonstrated that combining synthetic training data with real data can significantly improve the accuracy of deep learning-based image analysis methods^73–77^.

Despite these promising advancements, several challenges and bottlenecks are associated with synthetic data^78^ generated by AI-based generative models. Being purely data-driven methods, those models are in general limited by the quality and diversity of the training data^70,73–75,77^. While they are capable of interpolating within the distribution of the training data effectively, their ability to extrapolate beyond this distribution is often limited, potentially leading to less accurate synthetic data^79^. Another critical challenge is the potential introduction of bias, as synthetic data generated from biased real-world datasets can perpetuate or even exacerbate these biases in AI models^79^. In addition, generative models typically cannot produce ground truth labels in the form of segmentation masks for the generated images^66^, which, however, are essential for image segmentation tasks where precise pixel-level accuracy is crucial. Ensuring the interpretability and transparency of generative models is also a complex task, especially in the context of ethical and regulatory considerations. Addressing these challenges is essential for the effective and responsible use of synthetic data in biomedical image analysis.

An alternative approach to creating photo-realistic synthetic training data which can mitigate some of the drawbacks associated with AI-generated data is the use of computer graphics methods^75,80^. For example, computer graphics methods allow for controlled variability in synthetic datasets, enabling the generation of a wide range of scenarios and conditions, which can improve the robustness and generalization capabilities of deep learning models^74,75,80^. Another key advantage of computer graphics techniques over AI-based generative models is the ability to generate ground truth labels, such as segmentation masks, alongside synthetic images^80–82^. This makes them highly suitable for creating training data, especially in tasks requiring precise pixel-level annotations. In the context of biomedical image analysis, computer graphics methods may offer the potential to simulate complex tissue structures, variations, or other histological features with high accuracy^83,84^, This can be particularly useful for augmenting limited real-world datasets and addressing the inherent variability in biomedical images. These methods also enable the incorporation of domain-specific knowledge, ensuring that synthetic data is both realistic and relevant to the specific application^81,84^.

In this work, we introduce SYNTA (synthetic data) as a novel computer graphics-based approach for the photo-realistic generation of highly complex synthetic biomedical images as training data for a state-of-the art segmentation network in the context of digital pathology. As a proof of concept, we performed a comprehensive quantitative assessment of images from skeletal muscle tissue sections derived from desmin knock-out (DKO) mice^85–87^, a well-established animal model for human autosomal-recessive desminopathies with lack of desmin^88^. For a diagnostic evaluation of skeletal muscle specimens, number, size and form of muscle fibers give important basic information allowing classification of the sample in either healthy or diseased^89^. Manually segmenting muscle fibers or measuring the fiber diameter is a time-consuming process, usually limited to small sample sizes (~400 fibers)^90,91^ and is prone to human variance. We show that our approach, which does not rely on AI-based generative models but is solely based on interpretable parametric texturing and computer graphics, can be used to train a robust fiber segmentation network to predict expert-level results on previously unseen but real-world data. Furthermore, we demonstrate the model’s superior generalization capabilities compared to a network that was trained on real data. Finally, we analyze over 208,000 muscle fibers from a real-world dataset based on our approach, with the fully automated analysis taking approximately 5 min per entire tissue section. Given the large number of quantitatively analyzed muscle fibers, we show that even a single fiber feature (diameter) is sufficient to distinguish between control and pathology induced tissue samples.

## Methods

### Biomedical image segmentation using photo-realistic synthetic data

**Fig. 1:**
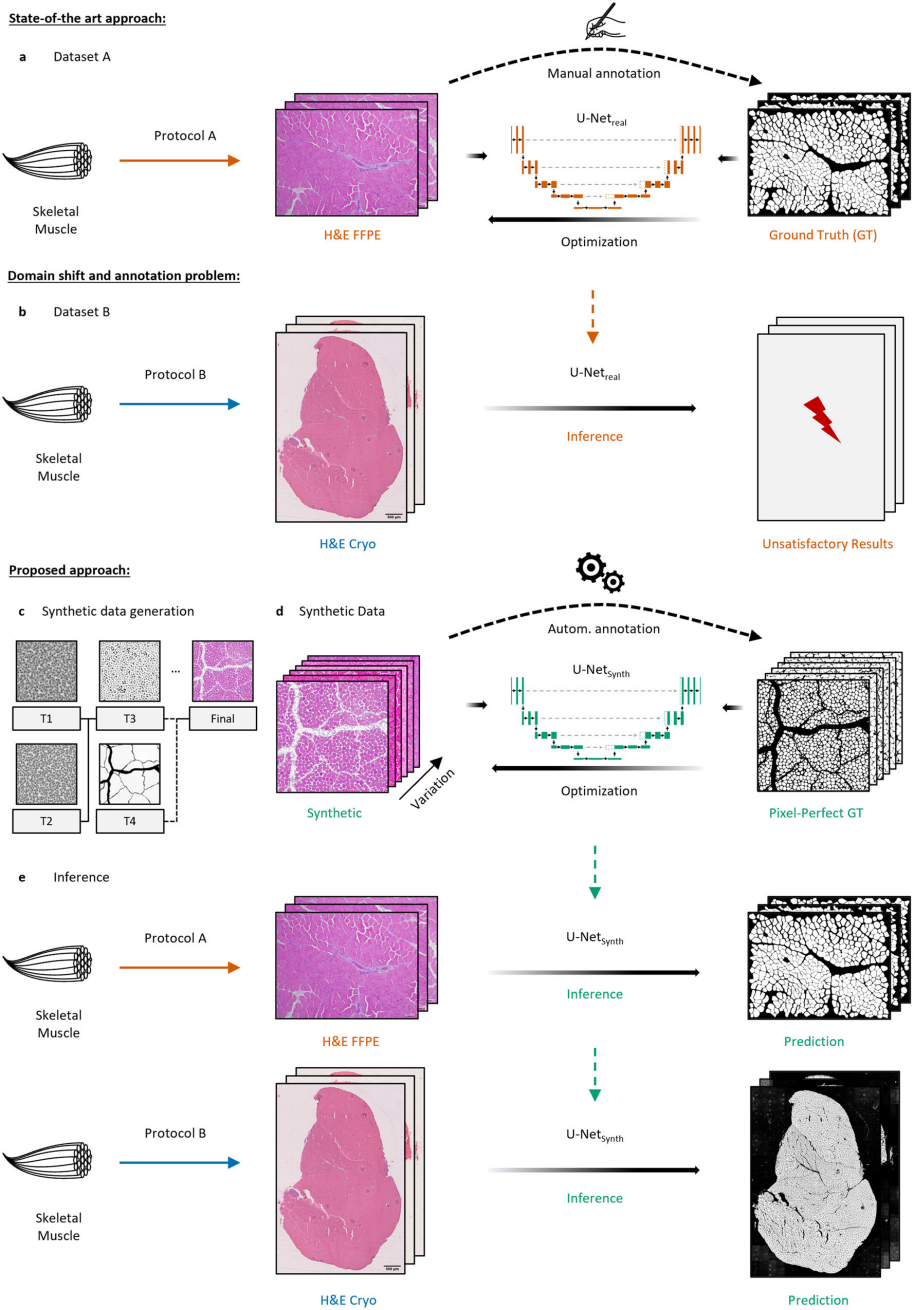
Illustration of the state-of-the art approach for the segmentation of biomedical image data, the problems that are associated with this approach and the proposed methodology (SYNTA) using photo-realistic synthetic training data. **a** The classical approach for DL-based biomedical image segmentation requires the manual annotation of a (specialized) dataset to train a model (U-Net_real_) on the fiber segmentation task. **b** As sample preparation protocols or the acquisition hardware can change during experiments or among institutions, the difference in distributions between the two datasets A and B can lead to the domain shift resulting in poor generalization of U-Net_real_ across the datasets. **c** Illustration of the SYNTA data generation pipeline. Pre-defined parametric textures (T1–T4) were used to create a simulation pipeline that imitates the inherent features (fiber shapes, tissue artifacts, staining variance, etc.) of H&E-stained normal murine skeletal muscle brightfield images. **d** Based on the simulation, a photo-realistic and highly diverse synthetic dataset containing over 74,000 muscle fibers with its pixel-perfect GT labels was generated and used to train a segmentation model (U-Net_synth_) on the synthetic data only. **e** Although solely trained on synthetic data, U-Net_synth_ was not only able to predict expert-level segmentation on previously unseen real-world data, but it also generalized well on both datasets A (H&E-stained sections of formalin-fixed paraffin-embedded skeletal muscle tissue (H&E FFPE)) and B (H&E-stained sections of cryo-preserved skeletal muscle tissue (H&E Cryo)).

Following the supervised learning paradigm, to train a state-of-the art CNN (U-Net^92,93^) segmentation network, a considerable number of images have to be annotated manually (Fig. 1a). However, as sample preparation protocols and data acquisition hardware may vary during experiments and among institutions, the differences in the resulting datasets often lead to the *domain-shift*^44^ causing poor segmentation performances of U-Net_real_ on the new dataset B (Fig. 1b). To reliably address this problem, in general a refinement of the model on the new dataset is needed by adding a significant number of representative and manually annotated new samples to the training data.

In this work, we propose an alternative solution to this problem with SYNTA. We used real-world images as visual reference and hand-crafted a pipeline of parametric textures (Fig. 1c, T1–T4) in a 3D computer graphics software (the open-source software Blender^94^) to generate the inherent features (fiber shapes, connective tissue and nuclei alternations, artifacts, staining variance, etc.) present in H&E-stained skeletal muscle brightfield images (see Supp. Fig. 1a). Once the SYNTA simulation pipeline was implemented, this fully interpretable simulation approach allowed us to automatically render a highly diverse photo-realistic synthetic dataset containing over 74,000 muscle fibers and its respective pixel-perfect labels. We then trained a U-Net (U-Net_synth_) on the synthetic data only (Fig. 1d). After the training procedure, U-Net_synth_ was not only able to perform well on previously unseen real-world data, but the diversity within the synthetic dataset also resulted in U-Net_synth_ to generalize well across the datasets A and B, resulting in expert-level fiber segmentation quality for both real datasets (Fig. 1e). A refinement of U-Net_synth_ on real data was not necessary.

### H&E FFPE (formalin-fixed paraffin-embedded) sample preparation and staining protocol

Muscles of male C57BL/6J wild-type mice were isolated at 15 weeks of age, fixed for overnight in phosphate-buffered 4% formaldehyde (Roti-Histofix, Carl Roth GmbH) and stored in 70% Ethanol for several days. Muscles were embedded in paraffin and 2 µm sections were cut and stained for H&E.

Paraffin slides were dewaxed by applying Xylol two times for 5 min each at room temperature (RT). Then slides were immersed in 100% isopropyl alcohol for 2 min twice followed by two times immersion in 96% isopropyl alcohol for 2 min. Deionized water was used to wash the slices. Mayer*’s* hemalum solution (Merck 1.092490500) was mixed 1:10 with deionized water and slides were immersed in the resulting solution for 10 min at RT. Slides were washed with deionized water, followed by washing with 1% HCl-ethanol. Then slides were exposed to running tap water for 10 min at RT. Slides were immersed in Eosin solution (Sigma) consisting of 0.5% Eosin and 0.01% acetic acid in deionized water and left in this solution at RT for 15 min. Deionized water was used to wash the slides again, followed by immersion in an increasing ethanol series with a final brief exposure to EBE (Merck). Finally, slides were mounted using a xylol free mounting medium. The H&E-stained sections of FFPE muscle from male wild-type mice used in this work were prepared as part of other research projects. There was no need to include additional mice, as the sex of the mice has no effect on the identification of the basic morphological muscle parameters analyzed in this work. The use of pre-existing samples of male-only mice in the H&E FFPE dataset is therefore not relevant to the results of this project.

### H&E FFPE dataset (dataset A)

The H&E FFPE wild-type mouse dataset consisted of 12 expert-annotated images of the size 4140 × 3096 pixels of skeletal muscle, containing a total number of 6204 labeled muscle fibers. The image acquisition was performed using a Zeiss Axio Lab.A1 laboratory microscope and the cellSens Entry Software (OLYMPUS, version 1.3) at 10x optical zoom (Zeiss N-Achroplan 10x/0,25 Ph1 ∞/- objective) and a resolution of 0.34 µm per pixels (2.933 pixels per µm).

### H&E Cryo sample preparation and staining protocol

Gastrocnemius/soleus muscle groups were dissected from female DKO Hom and healthy WT mice controls at age of 28–34 weeks, embedded in Tissue-Tek OCT compound (Sakura Finetek), immediately frozen in liquid nitrogen-cooled isopentane, and cryostat sections of 6 µm thickness were collected on microscope slides, air-dried for 30 min, and used for H&E stains.

Cryosections were incubated in filtrated hematoxylin solution (Gill II, Epredia 6765007) for 2 min. Afterward, slides were rinsed in room temperature tap water, then incubated in tap water for 2 min, and immediately transferred into an eosin staining solution (Bio Optica, 05-100003/L) for 30 s. For dehydration, slides were briefly incubated in isopropanol (1 × 70%, 2 × 96%, 2 × 100%), and then transferred into xylene before mounting with a coverslip. The H&E-stained cryosections of muscle from female homozygous DKO mice and wild-type siblings used in this work were prepared as part of other research projects. There was no need to include additional mice, as the sex of the mice has no effect on the identification of the basic morphological muscle parameters analyzed in this work. The use of pre-existing samples of female-only mice in the H&E Cryo dataset is therefore not relevant to the results of this project.

### H&E Cryo dataset (dataset B)

The H&E Cryo desminopathy mouse dataset consisted of 27 WSI images of varying image sizes from soleus/gastrocnemius muscle sections of 4 DKO Hom mice and 5 WT siblings. WSIs were acquired using a Hamamatsu NanoZoomer S60 (C13210) whole slide scanner at 40× magnification with a scanning resolution of 0.22 µm per pixels. From the 27 WSI, we manually extracted 52 images of single tissue sections (28 DKO Hom, 24 WT) at a resolution of 0.6615 µm per pixels (1.5117 pixels per µm) using QuPath^95^ and Fiji^96^.

### Simulation of skeletal muscle bright-field microscopy images

**Fig. 2:**
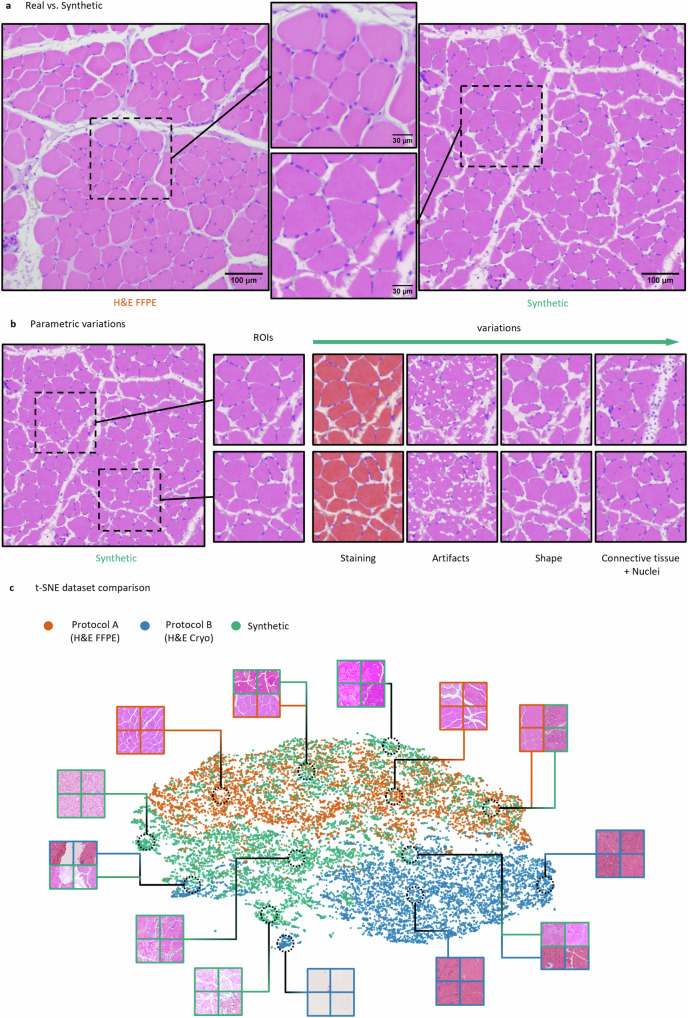
Direct comparison of synthetic and real data. **a** Exemplary real image of a H&E FFPE (dataset A) stained normal murine muscle tissue acquired using a brightfield microscope (left) in comparison to a photo-realistic synthetic image (right) generated using our simulation approach. **b** Example parametric variations within a single synthetic image. The pipeline allows to control every feature of the synthetic image to create realistic variations. This includes a diversity of the staining, sizes, shapes, positions and densities of muscle fibers, nuclei, and connective tissue components. **c** t-SNE plot comparing the three datasets. Each data point represents a randomly extracted image patch of the size 256 × 256 pixels. In total, 6000 image patches were extracted for the t-SNE visualization for each dataset, respectively.

The simulation of photo-realistic skeletal muscle bright-field microscopy images is based on texturing techniques, which allow to create fully parametric simulations through combinations of textures. This enables the generation of large randomized digital content by deterministic and human pre-defined parameters. During the content generation process, the texture parameters are sampled from a (pre-defined) range of values following specific design rules, such that the computed output of the pipeline resembles realistic variations. In this work, we used this technique in combination with the open-source 3D software Blender^94^ (version 2.93) to render realistic microscopic images of skeletal muscle tissue. To do so, we first visually inspected the features (fiber and nuclei shapes and coloring, tissue artifacts, connective tissue etc.) of real-world reference images to gain sense of the diversity within such images. Afterward, we used the rendering software to hand-craft a comprehensive texturing pipeline, which was mainly based on the Worley noise algorithm^97^ in order to imitate a wide range of the observed variations (see Fig. 2b, Supp. Fig. 1). After the implementation of the pipeline, we pre-defined a set of deterministic parameter ranges to ensure that the generated results resemble a diverse but realistic variety of synthetic skeletal muscle images.

### Synthetic dataset

Based on the parametric texturing pipeline, we automatically generated 120 synthetic images of the size 2048 × 2048 pixels using the Python application programming interface of Blender. During this data generation process, various random variations were introduced (see Fig. 2b) to alter the appearance of the muscle fibers, nuclei, connective tissue (perimysium and endomysium) and the background. These included variations for the staining, size, shape, and distribution of fibers and nuclei as well as diverse changes for the connective tissue and tissue artifacts. For each rendered image, a corresponding ground truth fiber segmentation mask was automatically extracted from the pipeline. In total, the synthetic dataset contained ~74,000 muscle fibers with their respective pixel-perfect GT annotations. Based on fiber size comparisons of the synthetic dataset with real data, the pixel resolution distribution within the synthetic dataset was 0.79 ± 0.22 µm per pixel.

### Dataset comparison

Three datasets (H&E FFPE, H&E Cryo and the synthetic dataset) were compared via t-SNE^98^ as illustrated in Fig. 2. The t-SNE visualization is based on image features extracted by a VGG16^99^, which was pre-trained on the ImageNet^100^ dataset. As input for the VGG16, we used 6000 random image patches of the size 256 × 256 pixels from each dataset. To further reduce the dimensionality of each feature vector, we applied a principal component analysis^101^ (PCA) while covering 90% of its variance. Afterward, t-SNE with a perplexity of 50, 1000 iterations, a learning rate of 50 and an Euclidean distance metric was used to visualize all data points. The t-SNE algorithm was applied using the scikit-learn^102^ (version 0.24.0) Python machine learning framework.

### U-Net specifications and training

Both U-Nets^93^ (U-Net_real_ and U-Net_synth_) share the same three-class architecture predicting background, fibers and boundary pixels as segmentation masks, while only the fiber segmentation masks were considered for the analysis. The input of the networks was a three-channel (RGB) image. As network architecture, we used the specifications proposed by the original U-Net publication^93^. In addition, we used Group Normalization^103^ as normalization techniques during training and applied bilinear upscaling instead of the originally proposed deconvolutions in the decoder part of the network.

The training of both U-Nets (U-Net_real_ and U-Net_synth_) was performed on three-channel (RGB) image patches for 120 epochs using stochastic gradient descent and the Adam^104^ optimizer. As training parameters, we used cross-entropy loss, learning rate decay with an initial learning rate of 0.001 and a mini-batch size of one with 150 iterations per epoch. The image patch size was set to 400 × 400 pixels. For data augmentation, random 90° rotations, additive noise and Gaussian blur was applied in combination with random brightness, contrast, saturation, and hue changes of an image patch. Before feeding images to the network, the images were normalized as additional pre-processing step. To strengthen the focus of the networks on the fiber borders, a pixel-wise weight map was computed on-the-fly during the training process following the proposed approach from ref. ^93^. In this context, we found that setting the w_0_ parameter to a very high value of ~400 with σ = 5 pixels achieved the best results. To compensate for the high weighting of the border pixels, we additionally included a weighting for the fiber bodies by a factor of ~50 and additionally weighted background pixels by a factor of 10. Both networks (U-Net_real_ and U-Net_synth_) were trained using the same training parameters. The networks were implemented and trained using the PyTorch^105^ machine learning framework (version 1.10.0). The implementation was based on ref. ^106^.

U-Net_real_ was trained on the complete H&E FFPE dataset with 12 manually expert-annotated images of the size 4140 × 3096 pixels. In total, the dataset contained 6204 labeled muscle fibers. As the synthetic data contained approximately 3.3× times more pixel data and ~12× times more annotated fibers (synth: 74221, H&E FFPE: 6204), U-Net_real_ was trained in a leave-one-out cross-validation setup to assess its general segmentation performance. Thereby, early stopping was used to prevent the models from overfitting on the training data. In this regard, 9% of the training set served as validation data.

The training of U-Net_synth_ was performed on the entire synthetic dataset using the same training parameter as used for U-Net_real_. For the selection of the best performing U-Net_synth_ model, we followed the model selection approach as proposed by Mill et al.^81^: after each epoch the model performed a segmentation on real (H&E FFPE) validation image patches of the size 1000 × 1000 pixels. Afterward, the segmentation performance was assessed quantitively for each prediction. Based on this evaluation, the model with the best performance was chosen for the segmentation of all real (H&E FFPE and H&E Cryo) data.

### Benchmarking

The segmentation performance of U-Net_real_ and U-Net_synth_ was compared to the generalist cytoplasm model of Cellpose^43^. Cellpose is a state-of-the art generalist segmentation method which was trained on a dataset of varied cell images containing over 70,000 manually segmented objects, including images of muscle fibers. For the benchmarking on the H&E FFPE dataset, we used the official implementation from ref. ^43^ with an object diameter size of 120 pixels and the default flow threshold of 0.4.

### Quantitative evaluation on H&E FFPE data

**Fig. 3:**
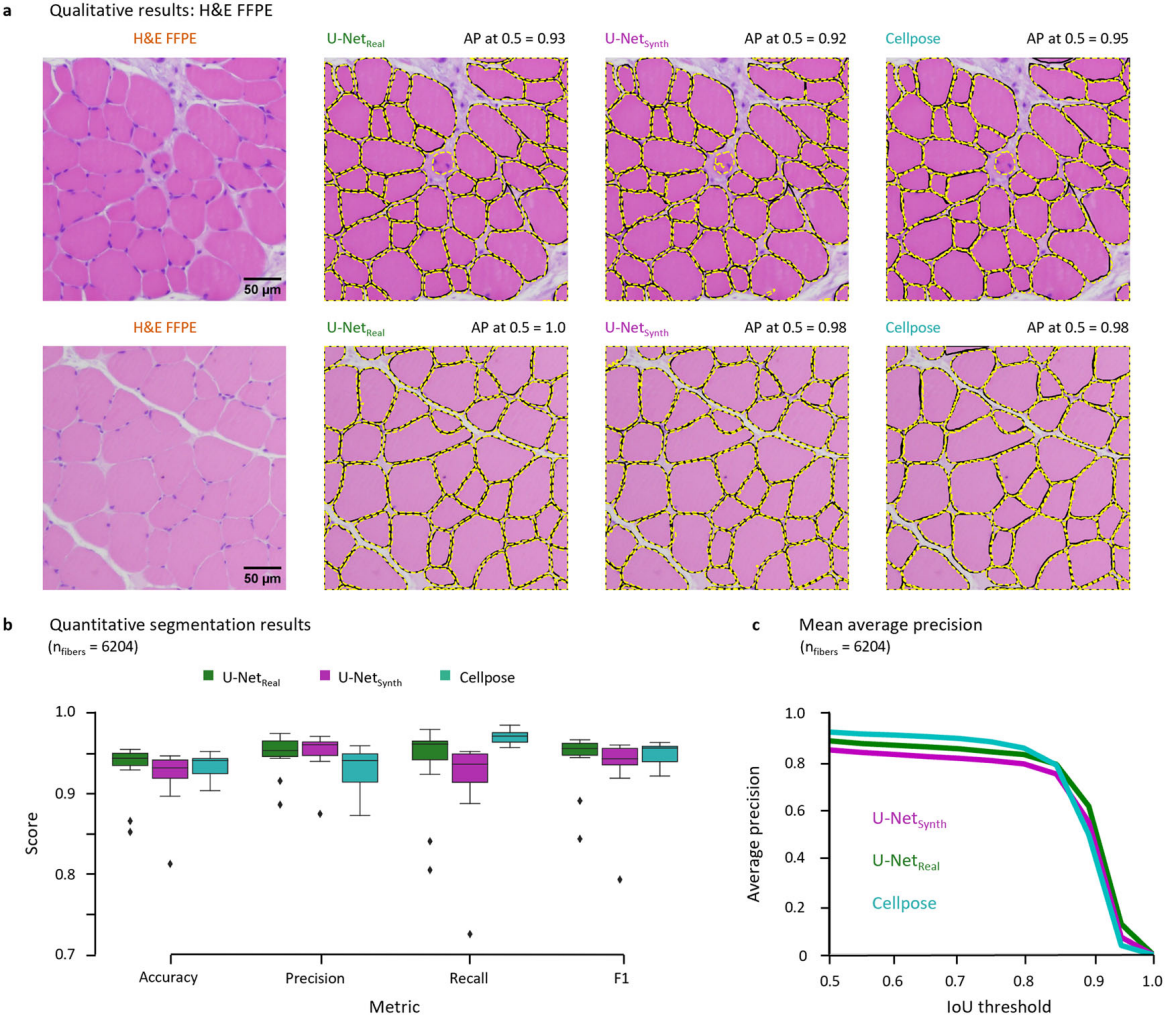
Segmentation results on the H&E FFPE data (dataset A). **a** Qualitative segmentation results of U-Net_real_, U-Net_synth_ and Cellpose on two example test images. The black fiber contours indicate the manual GT annotations, the dashed yellow lines denote the model predictions. The average precision (AP) at an IoU of 0.5 is given for each model above the respective images. **b** Comparison of the quantitative segmentation results for the H&E FFPE dataset based on the accuracy, precision, recall and F1 metrics. **c** Instance segmentation performance based on the mean AP for a IoU threshold in the range of [0.5, 1] in steps of 0.05.

The model performances were quantitatively evaluated on the H&E FFPE dataset using 6204 manually annotated ground truth fiber masks as baseline. For each prediction the accuracy, precision, recall, and F1 score^107^ was computed and averaged over all images. In addition, to assess the segmentation quality on a per-object basis, we quantified the overlap between a predicted and its corresponding GT fiber mask using the mean average precision^108^ (AP) as instance segmentation metric. In this context, we provide the AP for varying IoU thresholds in the interval of [0.5, 1.0] in steps of 0.05 (see Fig. 3c).

### Expert survey

**Fig. 4:**
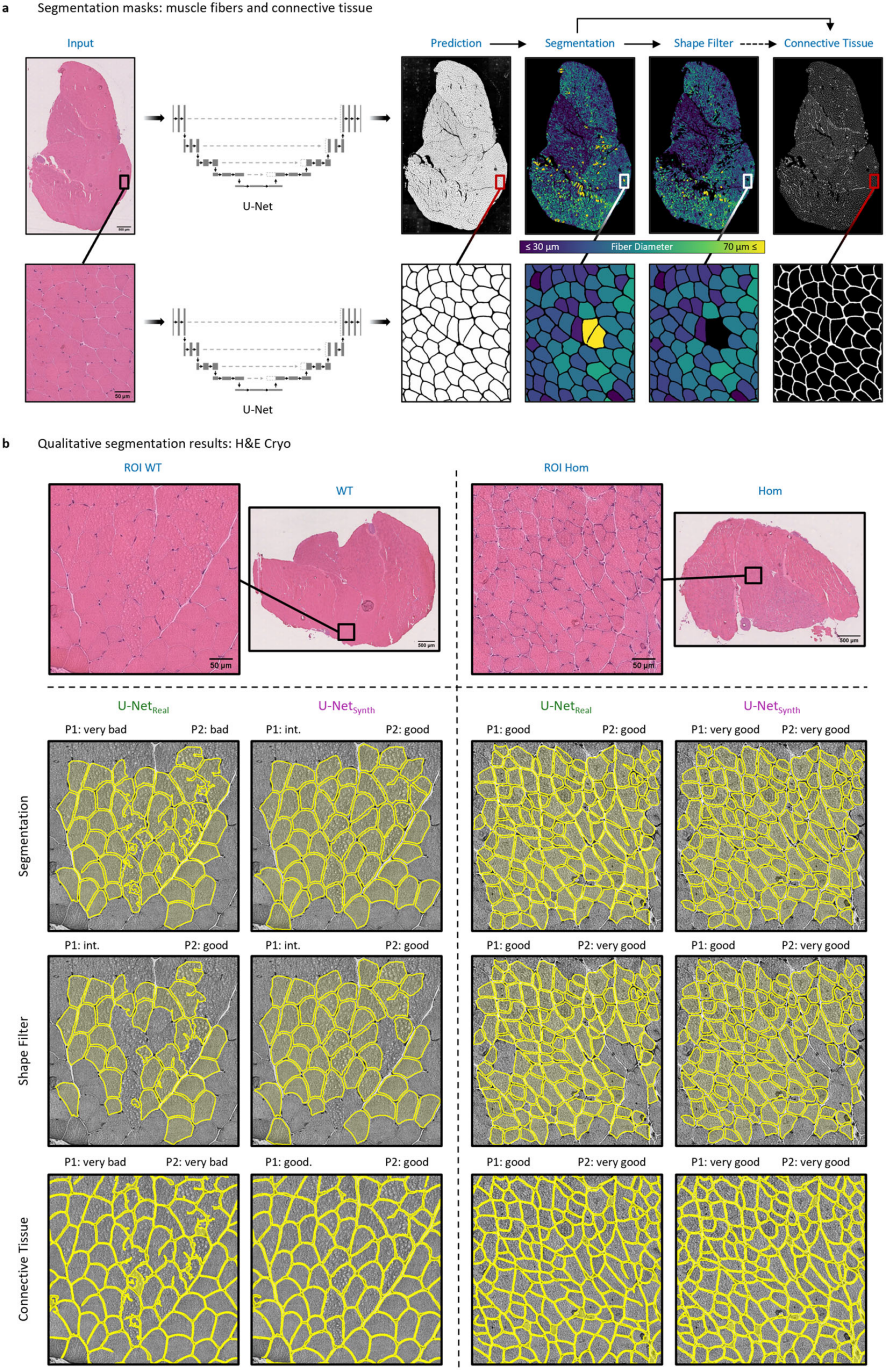
Segmentation results on the H&E Cryo data. **a** Model prediction based segmentation masks (“Segmentation”, “Shape Filter” and “Connective Tissue”) for an exemplary selected H&E Cryo whole slide image (WSI) of normal soleus/gastrocnemius skeletal muscle tissue derived from a wild-type mouse (WT). **b** To assess the model performance of U-Net_real_ and U-Net_synth_ on the unlabeled H&E Cryo dataset, the three segmentation masks were rated by two experts (P1 and P2) according to an ordinal scale. The top row exemplary shows two H&E Cryo expert pre-defined ROIs with the respective qualitative segmentation results of U-Net_real_ and U-Net_synth_. The ROIs refer to normal and myopathic soleus/gastrocnemius skeletal muscle tissue derived from wild-type siblings (WT) and homozygous desmin knock-out mice (DKO Hom), respectively. The expert ratings for the ROIs are denoted above the predictions for each category (“Segmentation”, “Shape Filter” and “Connective Tissue”), respectively.

For the expert survey on the H&E Cyro dataset, three ROI per section have been defined by a pathology expert (one ROI in the M. soleus and two ROI within the M. gastrocnemius), resulting in total 152 ROIs for the experiment. The size of the ROI was set to 400 × 400 µm. For each ROI, the two pathology-experts (P1 and P2) ranked the segmentation masks “*Segmentation*”, “*Shape Filter*” and “*Connective Tissue*” (see Fig. 4) of U-Net_real_ and U-Net_synth_, respectively. In this context, to reduce and eliminate experimental bias, the survey was conducted as a blinded experiment: for every section, the segmentation results of Net_real_ and U-Net_synth_ were randomly masked as segmentation from model ‘A’ or ‘B’. To account for the pixel resolution differences among the training datasets of U-Net_real_ (H&E FFPE) und U-Net_synth_ (synthetic dataset), predictions of U-Net_real_ on the H&E Cryo dataset were obtained at a pixel resolution of 0.34 µm per pixels, while predictions of U-Net_synth_ on the H&E Cryo dataset were obtained using a pixel resolution of 0.6615 µm per pixels. In addition, for the U-Net_real_ and U-Net_synth_ predictions the same post processing was used. The expert survey as well as all manual annotation on the H&E Cryo dataset including the ROI definitions was carried out using Cytomine^109^, an open-source collaborative web environment.

### Statistical analyses

To assess potential significant differences between the ranked U-Net_real_ and U-Net_synth_ predictions within the expert survey, we performed a paired t-tests with a significance level of *α* = 0.05 for each category “Segmentation”, “Shape Filter” and “Connective Tissue” (*n* = 156 ROIs per category) and for each expert (P1 and P2), respectively. The t-tests were computed using SciPy^110^ (version 1.4.1), a Python scientific computing library.

### Post processing

A custom Fiji (version v1.53f51) toolset was used to post process the model predictions in a fully automated manner. It is publicly available under https://github.com/OliverAust/HE_Muscle_Seg. First, the predicted probability maps were scaled to a range of [0, 255] and an intensity threshold of 210 was set to obtain the raw binary segmentation masks. Next, the objects were further filtered using a minimum object size of 150 µm. In addition, to exclude apparent non-organic shaped fibers from the analysis, objects below a circularity threshold of 0.1 were removed. The resulting binary mask is referred to as “post processed” in the following. For H&E FFPE image evaluation only these post processed images were considered, while for H&E Cryo images the following 3 post processing steps were additionally performed.

#### Segmentation mask

Masks of the whole muscle slice were generated. This was realized by dilating the post processed images ten times, followed by ten erosion operations. To remove muscle mask errors, the Fiji^96^ function “Area Opening” from the plugin MorphoLibJ^111^ was applied with a maximum size of 3500 pixels². To finalize muscle mask generation, a size filter of 1,500,000 µm² was applied. The muscle masks were then applied on the post processed images to remove any muscle fragments outside the muscle slice. The resulting images were used in the evaluation of the segmentation.

#### Shape filtering

A shape filter was used on the post processed image to generate additional images with pathologically relevant fibers only. A minimum circularity of 0.35 and maximum circularity of 0.95 were used for shape filtering. Muscle masks were also generated from shape filtered image as they are required for the connective tissue generation.

#### Connective tissue

Creating the difference between the muscle mask and the minimal threshold, or the shape filtered muscle mask and shape filtered images yields the connective tissue of the respective image. To evaluate the connective tissue, a selection was created from the respective muscle mask and then applied to the connective tissue. The connective tissue fraction and total area was measured inside the muscle mask area. The post processing, shape filtering and connective tissue workflow is visualized and discussed in detail in Supp. Figs. 2–4.

### Ethics declaration

Health monitoring of all mice was done as recommended by the Federation of European Laboratory Animal Science Associations (FELASA). Mice were handled in accordance with the German Animal Welfare Act (Tierschutzgesetz) as well as the German Regulation for the protection of animals used for experimental or other scientific purposes (Tierschutz-Versuchstierverordnung). Male wild-type C57BL/6J mice were housed in isolated cages under germ free conditions in a standard environment with free access to water and food. Sample preparation was carried out in compliance with all ethical regulations for organ removals at the University of Erlangen and carried out as approved by the local animal ethic committees of the Regierung von Mittelfranken (reference number 55.2.2-2532-2-1073). Female homozygous desmin knock-out (DKO) mice B6J.129S2/Sv-*Des*^tm1Cba^/Cscl^85–87^ and their wild-type siblings were housed in isolated ventilated cages (IVC) under specific and opportunistic pathogen-free (SOPF) conditions in a standard environment with free access to water and food. All investigations were approved by the governmental office for animal care (Landesamt für Natur, Umwelt und Verbraucherschutz North Rhine-Westphalia (LANUV NRW), Recklinghausen, Germany (reference numbers 84-02.04.2014.A262 and 84-02.05.40.14.057)).

### Reporting summary

Further information on research design is available in the Nature Portfolio Reporting Summary linked to this article.

## Results

### Photo-realistic synthetic biomedical images

We based our simulation on the features of real skeletal muscle reference images to achieve the highest possible degree of photo-realism for the synthetic images (see “Methods” section). In this context, Fig. 2a shows a visual comparison of a real skeletal muscle image (left) of dataset A (H&E FFPE, H&E-stained sections of formalin-fixed paraffin-embedded normal murine skeletal muscle tissue) and a synthetic image generated using the proposed SYNTA concept (right). At this point we want to emphasize, that our simulation technique does not require any kind of real image data as input nor is it based on GANs.

Both, the real (left) and synthetic image (right) contain the typical features of microscopic brightfield images of H&E-stained cross-sections derived from skeletal muscle. These include e.g., muscle fiber shapes, peripheral nuclei, the endomysium, and perimysium components of the connective tissue as well as tissue artifacts. In addition, due to the parametric nature of our simulation, we were also able to introduce a considerable spectrum of realistic variations within the synthetic images in terms of the staining representation, fiber properties (densities, sizes, and shapes), the position and size of the connective tissue as well as the density and position of peripheral and central nuclei (Fig. 2b, Supp. Fig. 1).

To assess the similarity between the two real-world (H&E FFPE and H&E Cryo) and the synthetic datasets, the structure of the datasets was visualized in Fig. 2c by using a t-distributed stochastic neighbor embedding^98^ (t-SNE). For the visualization, 6000 image patches of the size 256 × 256 pixels were extracted from each dataset, respectively. The distribution of the H&E FFPE (orange) and H&E Cryo (blue) data points show well-separated clusters with only little overlap. In contrast, the synthetic data points (green) are scattered across the embedding space while showing intersections with both real-world datasets. This suggests an overall high degree of similarity between synthetic and real data points. However, the relatively strong overlap of H&E FFPE and synthetic samples may indicate a higher degree of similarity between these two datasets in comparison to the H&E Cryo data.

### Segmentation performance on the H&E FFPE data

We next wanted to assess whether the synthetic dataset was photo-realistic enough such that a state-of-the art deep segmentation network was able to operate on real data while being trained on synthetic data only. To test this hypothesis, we trained a U-Net (U-Net_synth_) on *n* = 120 synthetic images containing over 74,000 muscle fibers and compared its segmentation performance to a U-Net (U-Net_real_), which was trained on real H&E FFPE images (*n* = 13) with over 6200 expert-annotated fibers. Due to the comparably limited number of real H&E FFPE images, U-Net_real_ was trained in a leave-one-out cross-validation setup to assess its general segmentation performance. As additional benchmark, we compared the U-Net predictions to the performance of the generalist cytoplasm model of Cellpose^43^, which was pre-trained on a variety of cell and non-cell images, including images of muscle fibers and containing over 70,000 segmented objects^43^. As post processing routine for the U-Net predictions, we additionally implemented a fully automated Fiji^96^ macro toolset. A detailed description of the post processing steps, and other features of the toolset can be found in Supp. Figs. 2–4. The qualitative and quantitative segmentation results for the H&E FFPE datasets are displayed in Fig. 3. For the qualitative results, two exemplary test patches are shown in Fig. 3a. While the manual GT annotations are visualized as black contour lines, the fiber contours of the model predictions are indicated by dashed yellow lines.

For both test images (top and bottom row), all model predictions match the GT contour lines almost perfectly. These results are also reflected by the average precision (AP) at an Intersection over Union (IoU) threshold of 0.5, which is denoted above each prediction. The quantitative results for the H&E FFPE dataset are visualized in Fig. 3b, c. Across all evaluation metrics U-Net_synth_ (accuracy = 0.92, precision = 0.95, recall = 0.91, F1 = 0.93) showed a similar performance compared to U-Net_real_ (accuracy = 0.93, precision = 0.95, recall = 0.94, F1 = 0.94) and Cellpose (accuracy = 0.93, precision = 0.93, recall = 0.97, F1 = 0.95). Also, the generalist model of Cellpose (AP_0.5_ = 0.93) marginally outperformed U-Net_real_ (AP_0.5_ = 0.89) and U-Net_synth_ (AP_0.5_ = 0.85) according to the AP at the commonly used IoU threshold of 0.5. The performance of U-Net_synth_ was in line with the results of U-Net_real_ and Cellpose, although U-Net_synth_ was trained on synthetic data only.

### Segmentation performance on the H&E Cryo data derived from wild-type and desmin knock-out mice

Based on the conclusive results of U-Net_synth_ on H&E FFPE data, we tested for the assumption that the proposed approach of using synthetic training data alone would lead to a more robust DL model. Hence, we further compared the generalization capabilities of both U-Nets (U-Net_real_ and U-Net_synth_) on the H&E Cryo dataset. However, as no large-scale manual annotations were available for this dataset, we conducted an expert survey to grade the predictions of both models. In a first step, two neuropathology experts (P1 and P2) pre-defined 156 analytically relevant regions of interest (ROIs) in *n*_*WSI*_ = 52 whole slide images (WSI). Afterward, for each pre-defined ROI, the two experts evaluated three different segmentation masks: the quality of a fiber segmentation mask “*Segmentation*” containing diagnostically relevant and non-relevant fibers, a shape filtered segmentation mask “*Shape Filter*” that should contain only diagnostically relevant fibers by removing fibers according to its specific shape features (e.g., based on their diameter) and the quality of the connective tissue segmentation that is based on the non-shape-filtered fiber segmentation (Fig. 4a). For the survey, P1 and P2 graded each segmentation mask according to an ordinal scale (i.e., “very bad”, “bad”, “intermediate” (int.), “good” and “very good”). In total, 156 ROIs were evaluated by P1 and P2, for each model (U-Net_real_ and U-Net_synth_) and each category “Segmentation”, “Shape Filter” and “Connective Tissue”, respectively.

Fig. 4b provides a qualitative comparison of the segmentation results of U-Net_real_ and U-Net_synth_ for an expert-pre-defined ROI in images of H&E-stained sections of cryo-preserved murine soleus/gastrocnemius skeletal muscle tissue. To test the performance of both U-Nets, not only normal muscle (healthy murine muscle from wild-type (WT) siblings) was used as input, but also myopathic soleus/gastrocnemius muscle from homozygous desmin knock-out mice (DKO Hom). The top row visualizes the post processed fiber segmentation masks, the second row displays the shape-filtered fiber masks, while the bottom row shows the connective tissue segmentations. Both models, U-Net_real_ and U-Net_synth_, were able to predict meaningful segmentations. However, the qualitative results show that U-Net_synth_ is not only equal to but indeed outperforms U-Net_real_ on the fiber segmentation task. It generalized well on the H&E Cryo images providing precise and very accurate fiber segmentations. In contrast, U-Net_real_ often failed to find distinct fiber contours resulting in over-segmented fibers, which were subsequently removed by the shape filter leading to considerably more missing fibers in this mask. In addition, U-Net_real_ failed to handle freezing artifacts well while U-Net_synth_ on the other hand demonstrated to be more robust against such artifacts, leading to more accurate fiber segmentations (Fig. 4b, ROI WT). Consequently, the connective tissue segmentation of U-Net_synth_ also shows more accurate results compared to U-Net_real_.

**Fig. 5:**
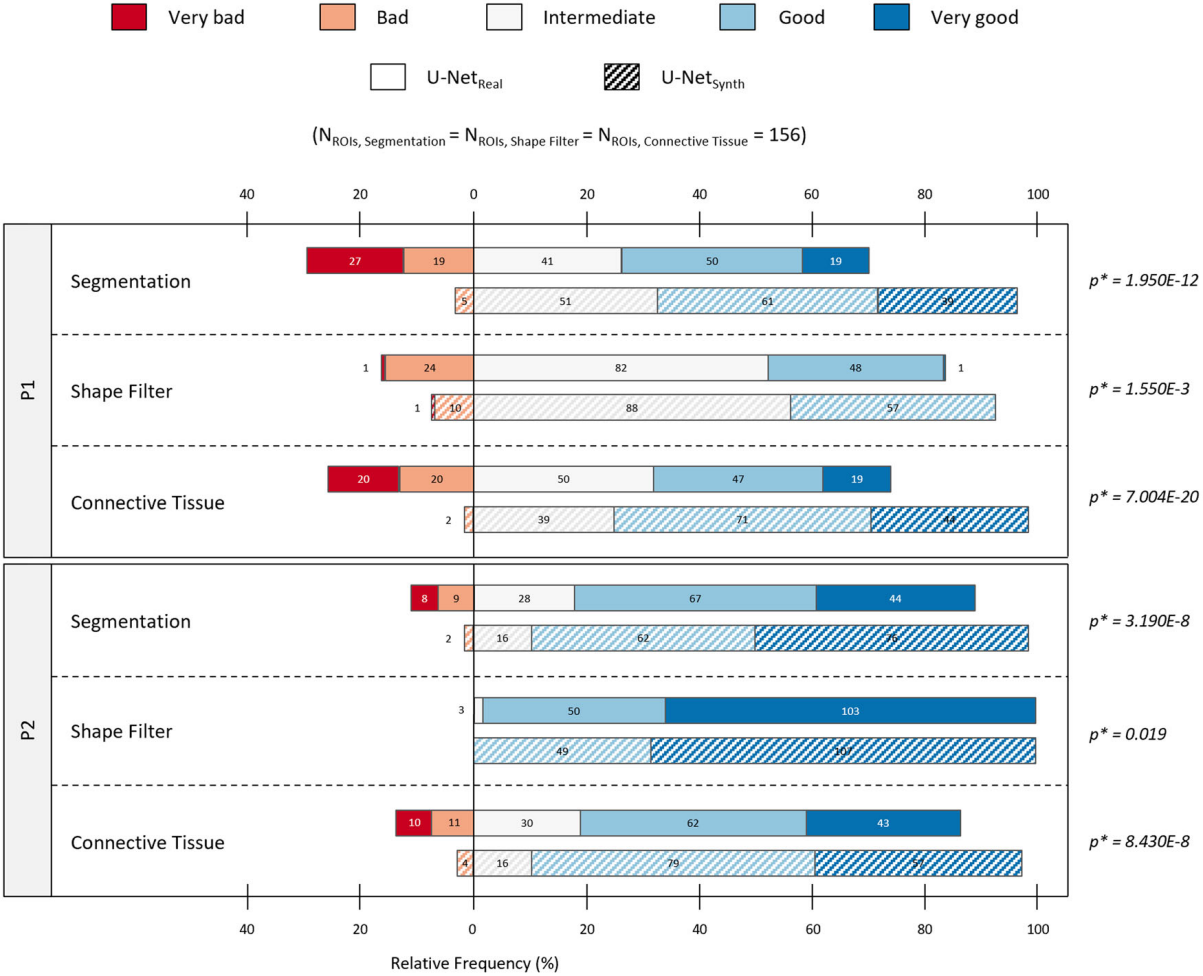
Expert survey results and the exclusion of regions with severe artifacts for the subsequent H&E Cryo dataset analysis. Diverging stacked bar chart showing the quantitative results of an expert survey evaluating the segmentation performances of U-Net_real_ and U-Net_synth_ on the unlabeled H&E Cryo dataset. Based on an ordinal scale each expert (P1 and P2) ranked the models’ predictions for 156 expert pre-defined ROIs for the categories “Segmentation”, “Shape Filter” and “Connective Tissue”, respectively. The absolute number and relative frequency of ROIs graded as “very bad” or “bad” are shown to the left of the zero line. The absolute number and relative frequency of predictions, which were ranked as “intermediate”, “good” or “very good” are visualized to the right of the zero line. Furthermore, to assess the significance of the ratings, for each category (“Segmentation”, “Shape Filter” and “Connective Tissue”) the *p*-values of a paired Student’s t-test are provided. In this context, the significance level was set to α = 0.05.

The quantitative results for the survey are provided in Fig. 5, which shows a diverging stacked bar chart^112^ based on the P1 and P2 expert ratings. According to the survey results, U-Net_synth_ predictions were consistently rated more favorably in all three categories. The results also show that predictions from U-Net_synth_ were in general considered as “Intermediate”, “Good” or “Very good” while in comparison the predictions from U-Net_real_ were rated “Bad” or “Very bad” more frequently. Overall, the U-Net_synth_ received significantly better ratings than the U-Net_real_ in all categories examined. To assess the significance of the ratings, a paired t-tests with a significance level of *α* = 0.05 for each category “Segmentation”, “Shape Filter” and “Connective Tissue” (*n* = 156 ROIs per category) and for each expert (P1 and P2) was performed, respectively. This means that according to the P1 and P2 evaluations, U-Net_synth_ significantly outperformed U-Net_real_ in all three categories, producing in general more accurate fiber and connective tissue segmentation masks. Based on these encouraging results, we used U-Net_synth_ to analyze the complete H&E Cryo dataset.

### In-depth H&E Cryo dataset analysis

**Fig. 6:**
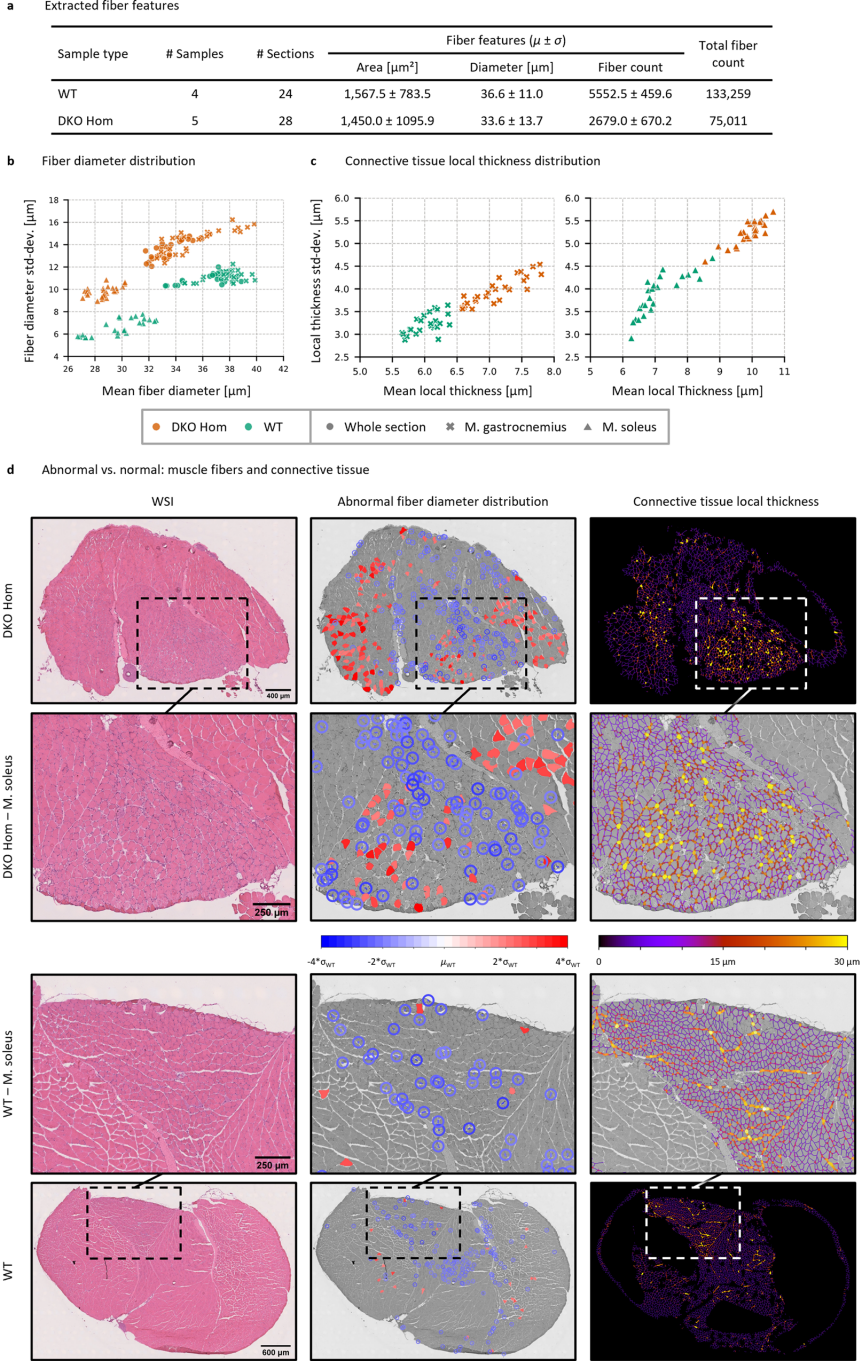
Analysis of the H&E Cryo dataset based on the U-Net_synth_ predictions. **a** Extracted fiber features from the H&E Cryo dataset. **b** Scatter plot of the fiber diameter distribution showing the mean fiber diameter (in µm) in relation to the fiber diameter standard deviation (std-dev.) (in µm). Each scatter point represents either a WT (healthy, wild-type sibling, in green) or a DKO Hom (abnormal/myopathic, homozygous desmin knock-out, in orange) section. Fiber diameters were analyzed considering either the whole section (circles), the M. soleus only (triangles) or the M. gastrocnemius only (crosses). **c** Scatter plots of the connective tissue local thickness distribution for each tissue section considering solely the M. soleus (right) or the M. gastrocnemius (left) parts. **d** Abnormal/myopathic vs. normal muscle fiber distribution and connective tissue based on the abnormality analysis.

To ensure an accurate analysis of the entire H&E Cryo dataset, we annotated specific regions within those sections that contained freezing artifacts (Type I) and artificial loosening (Type II) and subsequently removed such regions from the “Shape Filter” and the “Connective Tissue” segmentation masks (Supp. Fig. 5). From overall nine tissue samples (murine soleus/gastrocnemius skeletal muscle packages, 5 WT (healthy, wild-type siblings) and 4 DKO Hom (abnormal/myopathic, homozygous desmin knock-out) we obtained 52 WSI or sections (24 WT and 28 Hom sections) and analyzed a total of 208,270 muscle fibers (Fig. 6a). The mean computation time for the automated analysis per section was ~5 min (~2 min for the U-Net_synth_ predictions and 3 min for post processing and feature extraction). Desmin knock-out (DKO) mice^85,86^ are a well-established animal model for a rare autosomal-recessive form of human desminopathies with lack of desmin expression giving rise to skeletal muscle myopathy and cardiomyopathy^113^. Skeletal muscle tissue derived from these mice shows a myopathic pattern comprising an increased variation of fiber diameters and content of connective tissue that is most prominent in the soleus muscle^114^. To validate the performance of our approach in a disease context, we investigated whether it was possible to differentiate between WT and DKO Hom samples solely based on the features extracted from the U-Net_synth_ “Shape filter” and “Connective Tissue” segmentation masks. Note that the “Shape Filter” mask was used as the basis for the dataset analysis as the shape filter was optimized toward containing diagnostically relevant muscle fibers only (see Supp. Figs. 3 and 4). In addition, to investigate whether the phenotype was present differently in distinct muscle regions we manually annotated the musculus soleus (M. soleus) region for a subsequent differentiation of the analysis results between the whole section as well as the M. soleus and M. gastrocnemius parts.

The fiber diameters were analyzed considering either the whole section, the M. soleus only or the M. gastrocnemius only. In all three cases, WT sections could clearly be linearly separated from the DKO Hom sections (Fig. 6b). The fiber diameter distributions showed, as expected, a distinct shift toward smaller muscle fibers in DKO Hom thus leading to an increased diameter variance (see Supp. Fig. 6). In addition to the fiber diameter analysis, we further investigated the disease phenotype by addressing the local thickness of the connective tissue in either the M. soleus or M. gastrocnemius parts of the whole section.

Figure 6c shows the resulting scatter plots that display the mean local thickness (in µm) of the connective tissue for the M. soleus and M. gastrocnemius regions of each section, respectively. The results demonstrate that it is also possible to distinguish between WT or DKO Hom samples considering solely the local thickness of the connective tissue.

Based on the large number of analyzed WT muscle fibers, we next addressed the possibility to depict the general disease phenotype by assessing only local, specific regions in single sections by identifying abnormal fibers. In the context of the abnormality analysis, we defined a muscle fiber as abnormal if the difference of its diameter to the WT mean fiber diameter *µ*_WT_ was larger than |2σ|. Note that due to the differences of M. soleus and M. gastrocnemius in terms of mean fiber diameters (M. soleus fibers are in general smaller than M. gastrocnemius fibers, also see Supp. Fig. 6), M. soleus fibers were compared against *µ*_WT, M. soleus_, while M. gastrocnemius fibers were compared against *µ*_WT, M. gastrocnemius_.

Figure 6d displays all identified abnormal fibers in exemplary chosen DKO Hom and WT sections. For visualization purposes, abnormal fibers that were significantly smaller (blue) than *µ*_WT_ were encircled for a better visual identification. The analysis revealed that fibers of a small size in DKO Hom sections are mostly located in the M. soleus region with only a small number of fibers with increased diameter. In the M. gastrocnemius compartment close to the M. soleus part also many small fibers could be found. The above observed increase of the local thickness of the connective tissue was present in these areas with abnormal fiber size values. On the other hand, in the remaining part of the M. gastrocnemius both small and increased fibers were identified.

## Discussion

Here we introduced SYNTA as a concept for the generation of highly complex and photo-realistic synthetic histopathology images as training data for a state-of-the art segmentation network (U-Net). We validated our approach on the fiber segmentation in brightfield images of H&E-stained skeletal muscle sections for two different datasets of differently generated sample types and acquisition modes. We showed that the quality, the complexity, and the high degree of photo-realism of the synthetic dataset was sufficient to train a U-Net for the precise segmentation of muscle fibers in real-world images. Furthermore, a comparison of a U-Net trained on real data (U-Net_real_) with a U-Net trained on synthetic data only (U-Net_synth_) revealed that the performance of both models was not only similar, but U-Net_synth_ was able to generate expert-comparable fiber segmentation results on previously unseen real data. In addition, our experiments demonstrated the superior generalization capability of U-Net_synth_, which significantly outperformed U-Net_real_ on a second (but also real) dataset. We finally analyzed over 208,000 skeletal muscle fibers based on the expert-level performance of U-Net_synth_ and showed, that it is possible to distinguish between healthy control and diseased (desmin knock-out myopathy) tissue samples solely based on a single fiber feature or the thickness of the connective tissue.

Yet, while our findings indicate that U-Net_synth_ demonstrated a superior generalization capability over U-Net_real_, it is important to note that the assessment of the generalization capabilities and segmentation performances on the H&E Cryo data primarily relied on an expert survey only, which introduces subjectivity. In addition, U-Net_real_ was trained on a smaller (although real) dataset compared to U-Net_synth_. This disparity in dataset size could potentially limit the significance of the comparison between the two models, as an increased dataset size will in general lead to better generalization capabilities as demonstrated by Gu et al.^32^. Nonetheless, the issue of small dataset sizes with a low degree of representativeness and with a lack of a large number of high-quality annotations is still a prevalent challenge in the field. Limited and less diverse datasets can hinder the training of robust models, making our findings particularly relevant to real-world applications where data acquisition is often limited by practical and logistical factors. In this context, using the SYNTA approach allows to create more diverse datasets.

While in this work we could show that the SYNTA approach can lead to remarkable results, it is important to emphasize its differences to AI-based generative approaches such as GANs or DMs. With SYNTA being a purely knowledge-driven approach that relies on computer graphics methods only, aspects such as acquisition of vast amount of training data as well as the lack of interpretability—which are commonly associated with AI-based generative models—are bypassed. In this context, it is not necessary to acquire a significant amount of representative real-world images, as potential feature variations are parametrically incorporated within the simulation itself. Using computer graphics-based synthetic content generation provides the flexibility that is needed to incorporate realistic repeating image features and patterns, which are often observable in microscopic images (cells, nuclei, shapes, etc.), in interpretable and controllable simulations. Being a fully parametric approach, the proposed concept enables the integration of domain knowledge and human expertise directly in the data simulation process and thus the training data itself. This not only leads to more robust and unbiased DL models, but it also allows to incorporate specific image features that a DL model implicitly learns during training. In addition, the acquisition of high-quality manual annotations is bypassed since ground truth labels are automatically derived from the simulation process. Also, with SYNTA being a lightweight texture-based simulation only, no dedicated compute resources are required to generate a realistic dataset within a reasonable time frame. While using consumer-grade graphics cards may significantly accelerate the rendering process, the rendering time for a single realistic muscle image and its ground truth segmentation mask takes ~2 min only.

Still, complex challenges have to be faced when using computer graphics methods in the context of histopathology over AI-based generative models. One significant hurdle involves accurately simulating the intricate variations in tissue, cells, and textures that are characteristic of real histopathological images. Variability in tissue morphology, staining techniques, and sample preparation protocols can introduce a wide range of visual features that need to be replicated within the simulation. Capturing the diversity and subtle nuances of cellular structures and textures is essential for generating realistic and valuable synthetic datasets. Replicating this degree of complexity demands advanced expertise in computer graphics and may require considerable manual effort. However, compared to the manual effort of real-world data acquisition and labeling, this process still poses are reasonable and cost-effective alternative. In addition, ensuring that these synthetic images are realistic compared to actual histopathological samples may be critical for their use in training deep learning models. In this context, it may require domain-experts such as pathologists to determine the simulation parameters and validate the synthetic data in terms of realism and correctness.

With SYNTA being a proof-of-concept that is highly specialized for muscle histopathology images, our future work will explore its generalizability for applications within other domains and imaging modalities. While real-world biomedical images are very diverse and show a high degree of complexity, the image features often follow repeating patterns which can be represented by means of computer graphics methods. Hence, we are confident that this approach poses an interpretable and controllable alternative to state-of-the art image synthesis methods in biomedicine and histopathology. It may represent a first step to accelerate the development of quantitative image analysis solutions, increasing the level of representativeness of the generated synthetic datasets while also enhancing the interpretability of generative models.

## Supplementary information

Supplementary Information

Description of Additional Supplementary Files

Supplementary Data 1

Supplementary Data 2

Supplementary Data 3

Supplementary Data 4

Supplementary Data 5

Supplementary Data 6

Supplementary Data 7

Supplementary Data 8

Supplementary Data 9

Supplementary Data 10

Reporting Summary

## Data Availability

The source data that support the plots within this manuscript is included in the Supplementary Data: the source data for the plots in Fig. 3 is provided in Supplementary Data 1–2, the source data for the plots in Fig. 5 is provided in Supplementary Data 3–8, and the source data for the plots in Fig. 6 is provided within Supplementary Data 9–10. Additional data is available upon reasonable request to the corresponding author [L.M. and A.G.].

## Code availability

The Fiji toolset implementing the post processing procedure is available under https://github.com/OliverAust/HE_Muscle_Seg.
