# Supplementary material for: Deep learning-based image analysis in muscle histopathology using photo-realistic synthetic data
Source: Commun Med (Lond). 2025 Mar 6;5:64. doi: 10.1038/s43856-025-00777-y (PMC11885816; doi:10.1038/s43856-025-00777-y)
Supplement: Supplementary file 1 — Supplementary Information [file 43856_2025_777_MOESM1_ESM.pdf]

Deep learning-based image analysis in muscle histopathology using photo-realistic synthetic data

Leonid Mill[1, 2, *], Oliver Aust[3], Jochen A. Ackermann[3], Philipp Burger[3], Monica Pascual[3], Katrin Palumbo-Zerr[3], Gerhard Krönke[3], Stefan Uderhardt[3], Georg Schett[3], Christoph S. Clemen[4,5], Christian Holtzhausen[6], Samir Jabari[6], Rolf Schröder[6], Andreas Maier[2] and Anika Grüneboom[7, †]

[1] MIRA Vision Microscopy GmbH, 73037 Göppingen, Germany

[2] Pattern Recognition Lab, Friedrich-Alexander University Erlangen-Nürnberg (FAU), 91058 Erlangen, Germany

[3] Department of Medicine 3 - Rheumatology and Immunology & Deutsches Zentrum für Immuntherapie, Friedrich-Alexander University Erlangen-Nürnberg (FAU) and Universitätsklinikum Erlangen, 91054 Erlangen, Germany

[4] Institute of Aerospace Medicine, German Aerospace Center (DLR), Cologne, Germany

[5] Institute of Vegetative Physiology, Medical Faculty, University of Cologne, Cologne, Germany

[6] Department of Neuropathology, Universitätsklinikum Erlangen, Friedrich-Alexander University Erlangen-Nürnberg (FAU), 91054 Erlangen, Germany

[7] Leibniz-Institut für Analytische Wissenschaften - ISAS - e.V., 44139 Dortmund, Germany

[*] Lmill@mira.vision, [†] anika.grueneboom@isas.de

Supplementary Figures

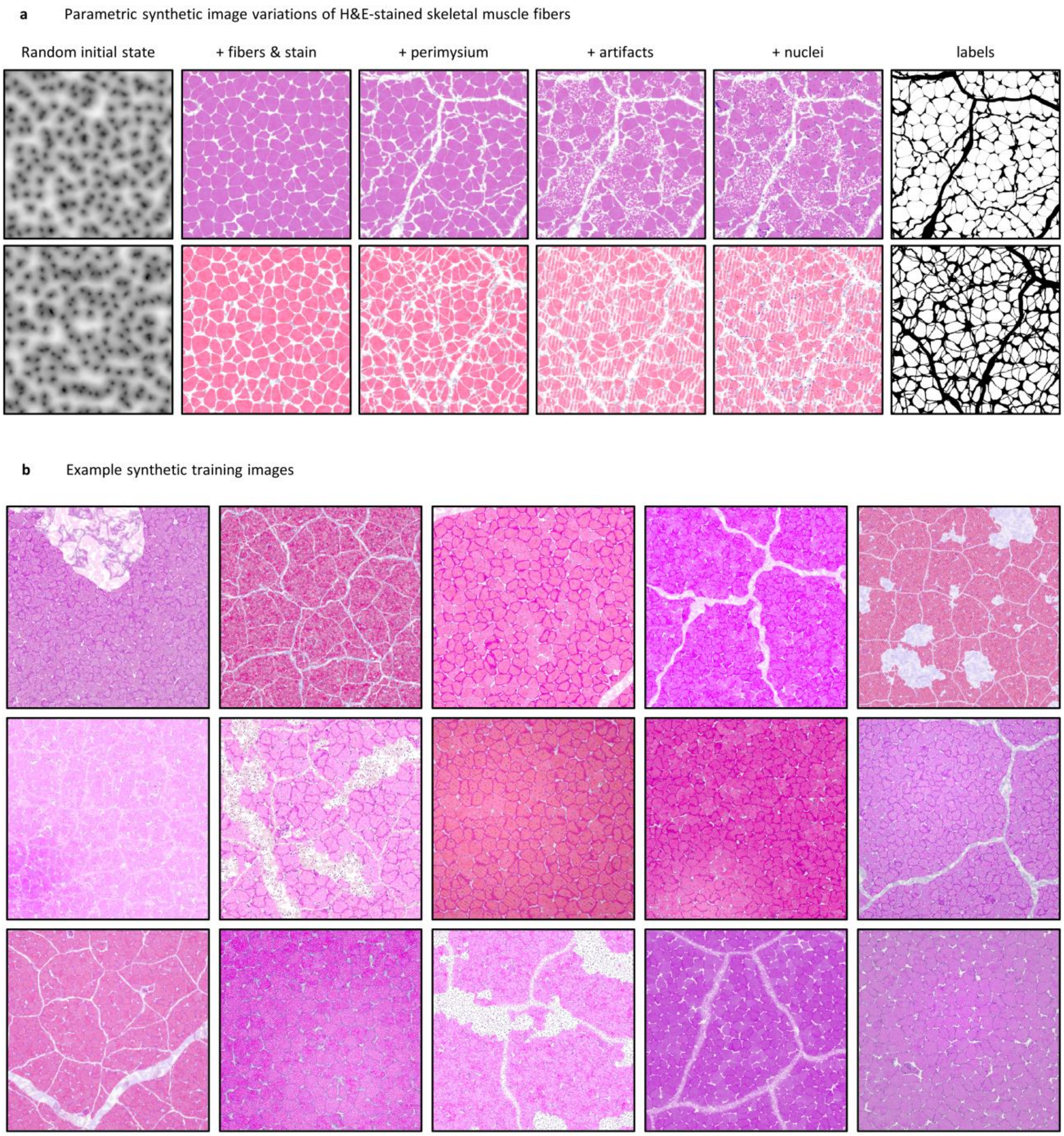

Supp. Fig. 1 | Parametric synthetic image variations of H&E-stained skeletal muscle fibers. a, Generation of realistic muscle fibers images. Based on a random initial state, randomized fibers, staining, perimysium, artifacts, and nuclei are added to obtain realistic renders while guaranteeing perfect segmentation labels with no annotation noise. **b,** Example realistic and non-realistic synthetic training images that were randomly sampled from the parametric image generation pipeline.

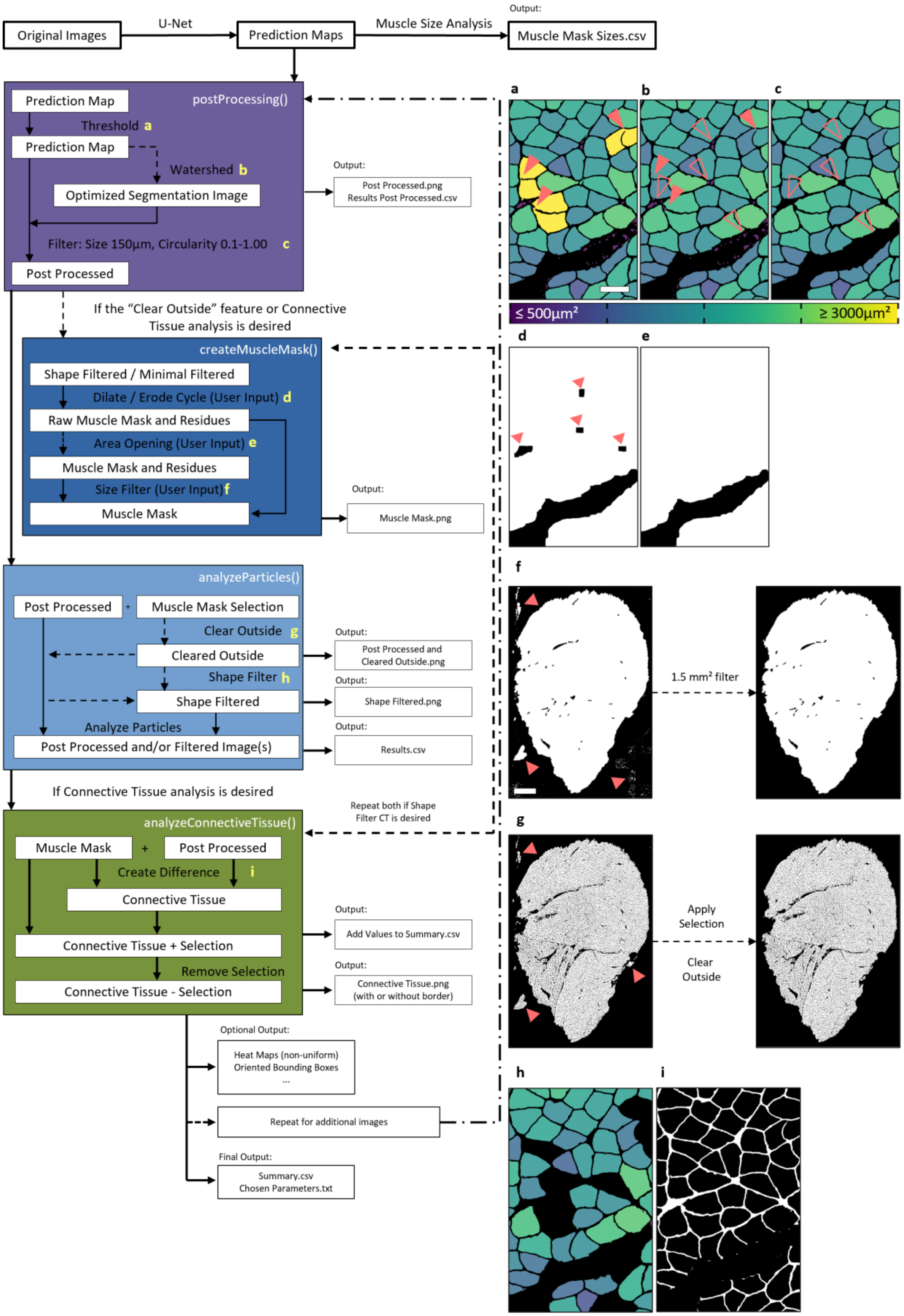
Original Images
U-Net
Prediction Maps
Muscle Size Analysis
Output:
Muscle Mask Sizes.csv
postProcessing()
Prediction Map
Threshold a
Prediction Map
Watershed b
Optimized Segmentation Image
Filter: Size 150µm, Circularity 0.1-1.00 c
Post Processed
Output:
Post Processed.png
Results Post Processed.csv
If the "Clear Outside" feature or Connective Tissue analysis is desired
createMuscleMask()
Shape Filtered / Minimal Filtered
Dilate / Erode Cycle (User Input) d
Raw Muscle Mask and Residues
Area Opening (User Input) e
Muscle Mask and Residues
Size Filter (User Input) f
Muscle Mask
Output:
Muscle Mask.png
analyzeParticles()
Post Processed
+
Muscle Mask Selection
Clear Outside g
Cleared Outside
Shape Filter h
Shape Filtered
Analyze Particles
Post Processed and/or Filtered Image(s)
Output:
Post Processed and Cleared Outside.png
Output:
Shape Filtered.png
Output:
Results.csv
If Connective Tissue analysis is desired
Repeat both if Shape Filter CT is desired
analyzeConnectiveTissue()
Muscle Mask
+
Post Processed
Create Difference i
Connective Tissue
Connective Tissue + Selection
Remove Selection
Connective Tissue - Selection
Output:
Add Values to Summary.csv
Output:
Connective Tissue.png (with or without border)
Optional Output:
Heat Maps (non-uniform)
Oriented Bounding Boxes
...
Repeat for additional images
Final Output:
Summary.csv
Chosen Parameters.txt
a
b
c
≤ 500µm²
≥ 3000µm²
d
e
f
1.5 mm² filter
g
Apply Selection
Clear Outside
h
i

Supp. Fig. 2 | Visualization of the Post Processing Workflow Diagram: The bold letters next to certain post processing steps in the diagram are represented by the image on the right side with associated bold letter. Functions of the Fiji Macro Code are indicated by a box with the function name on the top right to facilitate changes to the code. **a,** The original images are converted to prediction maps with the U-Net and subsequently a threshold is applied. Note that since fibers are merged the resulting area of that object is large and thus the fibers appear yellow. Higher thresholds can cause a higher area error but can also cause a smaller segmentation error. Three examples for segmentation errors are indicated by pink filled arrowheads. Please note that the various fibers area sizes are represented by a 'viridis' color map, ranging from 500µm² (dark purple) to 3000µm² (yellow). **b,** An optional watershed can segment such fibers efficiently as again indicated by the filled pink arrowheads. Additionally, the U-Net$_{synth}$ misinterprets some small tissue regions as muscle fibers (pink open arrowheads). **c,** These can be removed by a small size and circularity filter. The resulting images are referred to as "post processed" in this publication. To further improve analysis results and as a basis for connective tissue analysis a mask of the whole muscle section will be created. This is realized by several (e.g., 10) dilate operations that cause fiber-fiber gaps to be closed but also cause an enlargement of every structure. **d,** To keep the original outline of the whole muscle section, the same amount of erode operations are applied to the image. Completely filled holes and spaces are unaffected by the erode operations. Depending on the amount of dilate and erode operations applied, small holes will remain (pink filled arrowheads). **e,** These can be removed by inverting the image, using the area opening operation by MorphoLibJ [116] and inverting the image again. **f,** Finally, for whole slide images, a size filter must be applied to remove any unwanted muscle tissue fragments around the main muscle sample. **g,** The resulting mask of the whole muscle section is referred to as 'muscle mask'. This mask can be applied to the post processed image to remove any muscle tissue fragments outside the main sample that were left after the shape filter. **h,** Before the remaining objects in the image are analyzed and results are saved it is possible to apply a shape filter. The last step carried out is the analysis of the connective tissue. **i,** First, to create connective tissue images the difference between the muscle mask and the post processed image is generated. Since the gaps between the fibers are closed in the muscle mask image while they are still open in the post processed image, the difference of both images will be equal to fiber-fiber gaps. Local Mean thickness and the muscle mask selection is then used as the base to calculate connective tissue to fiber ratio. Shown images are for illustration purpose only. Scale bar **a**, 50 µm; Scale bar **f**, 500 µm.

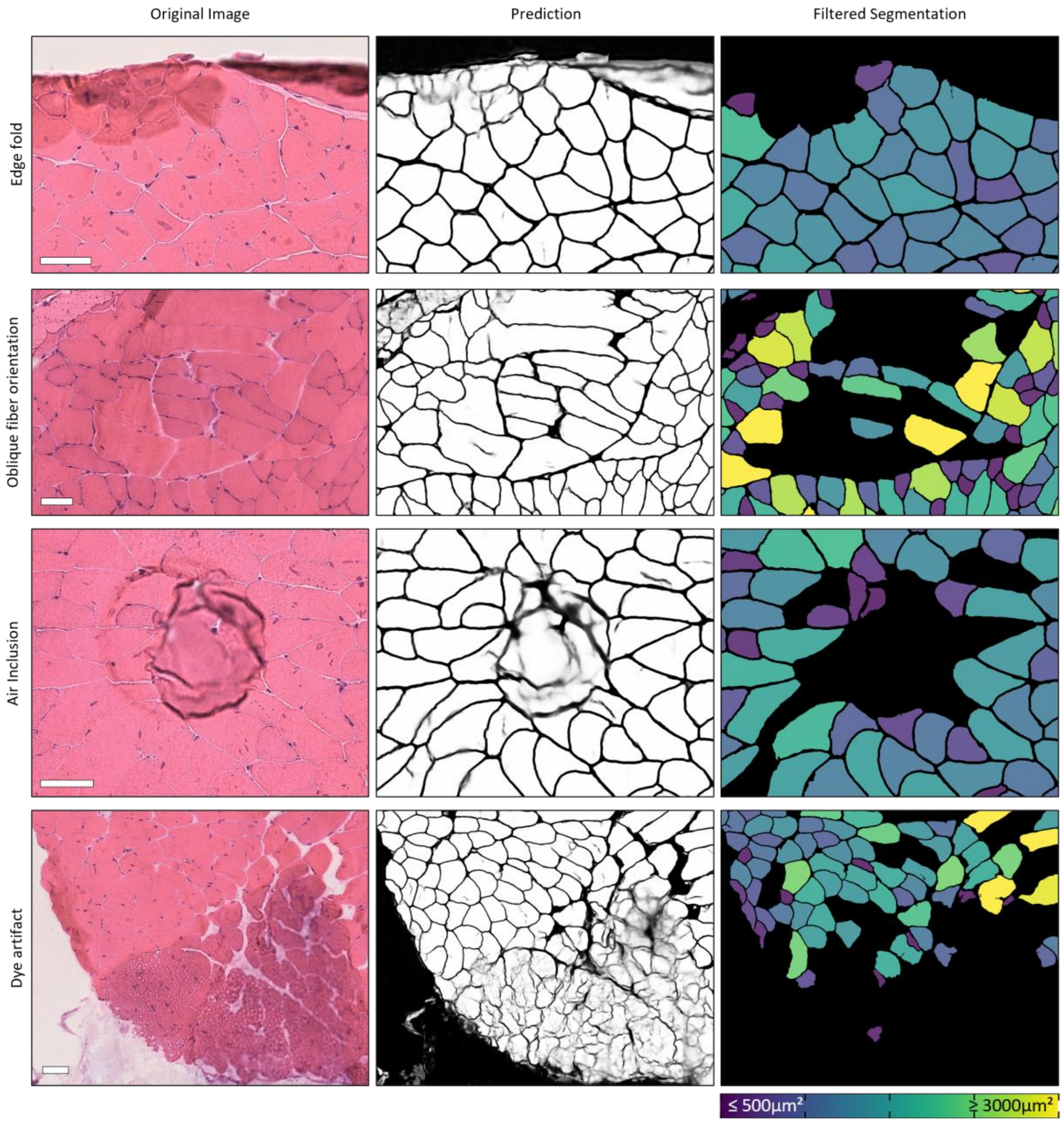

Supp. Fig. 3 | Artifact Handling during Post Processing. Sample preparation artifacts such as air inclusion and folded tissue (original unaltered images left column) can be misinterpreted by the U-Net as muscle fibers (predictions middle) since the fibers in artifacts still share many properties of normal muscle fibers such as form and coloring. As it is most likely that an inclusion of these fibers in the analysis is not desired, it is crucial to be able to remove them. With the use of shape filters such as circularity it is possible to remove most affected fibers (filtered segmentation right). Not all fibers can be removed as some artifacts have very fiber similar shapes. Since the type, occurrence rate and similarity to desired disease model observations will heavily depend on each individual project, these parameters can be adjusted accordingly in the available macro. The filter parameters used: Folded tissue: threshold set at 120, 15 maximum watershed, circularity filter of 0,45-1,00. Oblique and longitudinally oriented fibers within a cross section: threshold set at 140, 45µm maximum feret diameter filter, 0,4-1 circularity filter. Air inclusion: threshold set at 140, 0,45 – 1 circularity. Stain spilling: threshold set at 140, 0,4 – 1 circularity filter. Shown images are for illustration purpose only. Scale bars, 50µm. Please note that the various fibers area sizes are represented by a 'viridis' color map, ranging from 500µm² (dark purple) to 3000µm² (yellow).

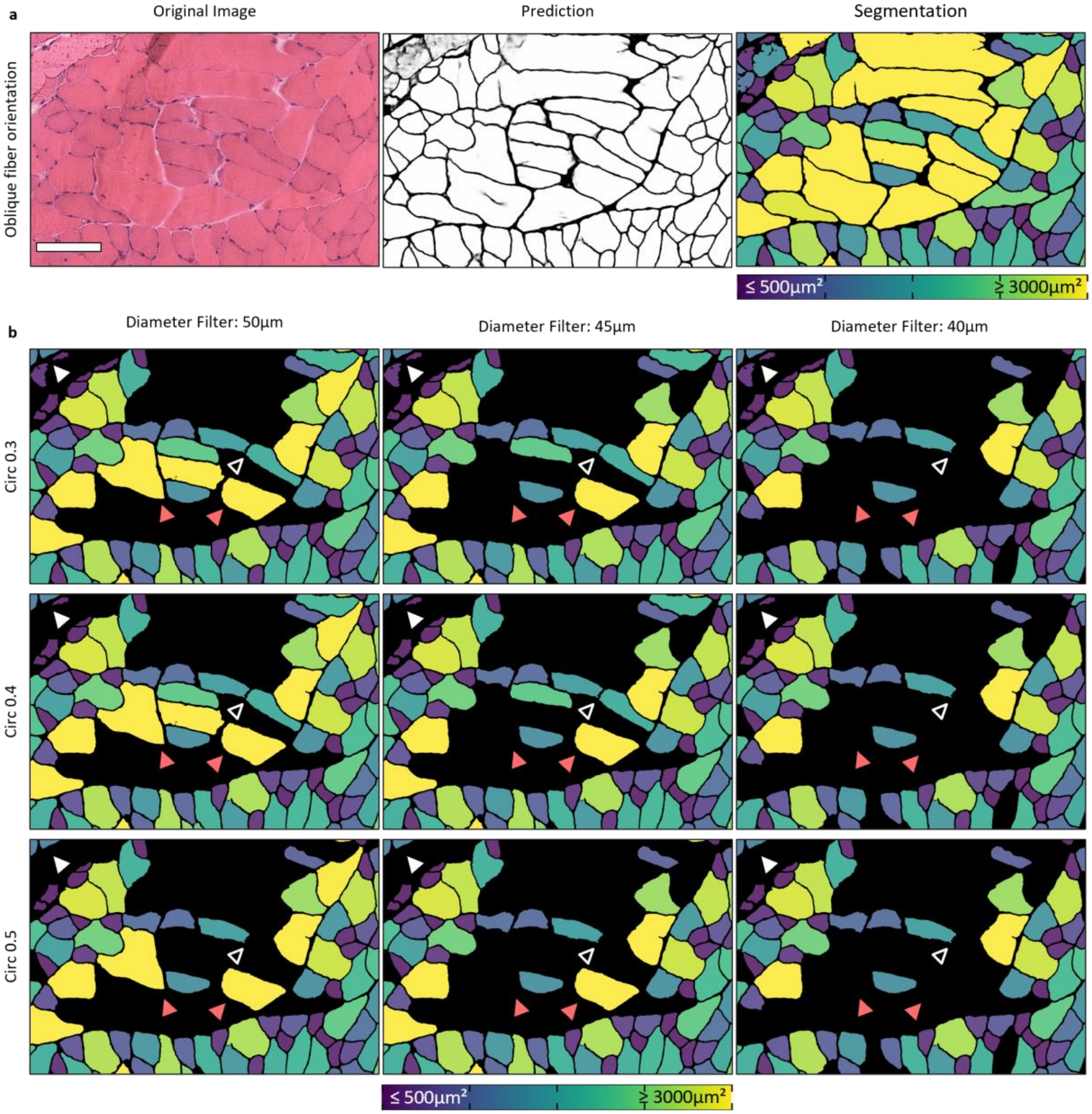

Supp. Fig. 4 | Visualization of the different shape filter parameters. a, shows the original image, the unprocessed prediction and the final segmentation. The depicted area of the cross section includes many oblique and longitudinally oriented fibers that users might want to exclude from their statistical analysis. **b,** shows the effect of the indicated shape filter parameter combinations in regard to the excluded fibers. It can be seen that some fibers can be excluded by both parameters (indicated with the white open arrowheads). Both a maximum Feret diameter and a minimal circularity filter can exclude these fibers. However, it can also be seen that some fibers can only be filtered by one of the parameters. For example, some fibers are only removed by setting a maximum Feret diameter while a circularity filter does not have any effect (magenta filled arrowheads). In contrary, smaller fibers originating from neural network misinterpretation (white filled arrowheads) are only removed by setting a circularity filter. Shown images are for illustration purpose only. Scale bar, 100µm. Please note that both in **a** and **b** the various fibers area sizes are represented by a 'viridis' color map, ranging from 500µm² (dark purple) to 3000µm² (yellow).

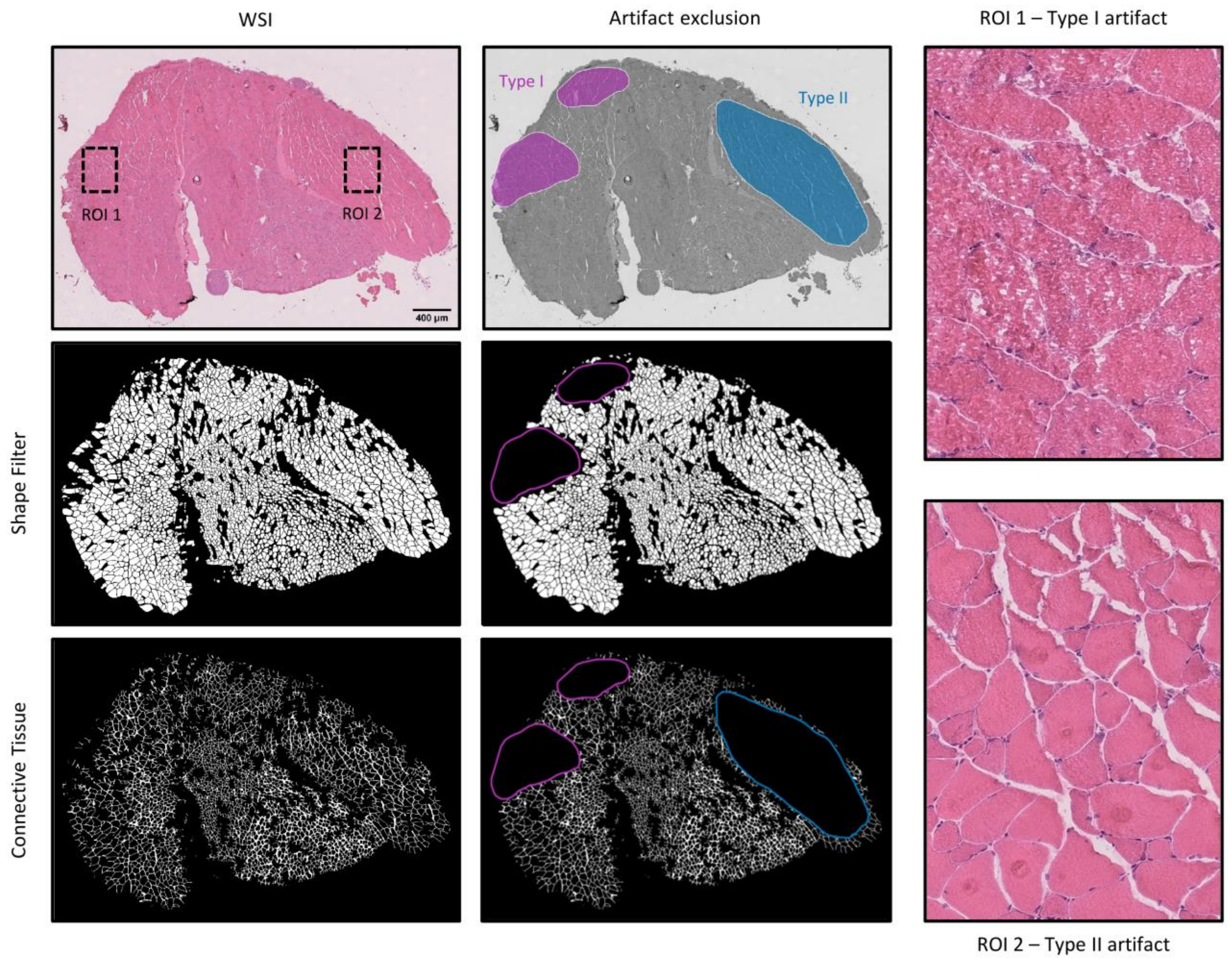

Supp. Fig. 5 | Exlusion of regions with severe artifacts from the analysis. For an accurate analysis of the H&E Cryo dataset, regions with marked freezing artifacts (Type I) or artificial loosening (Type II) were manually annotated. Fibers and connective tissue of the "Shape filter" and "Connective Tissue" that lied within such regions were automatically removed from the masks and excluded from the dataset analysis.

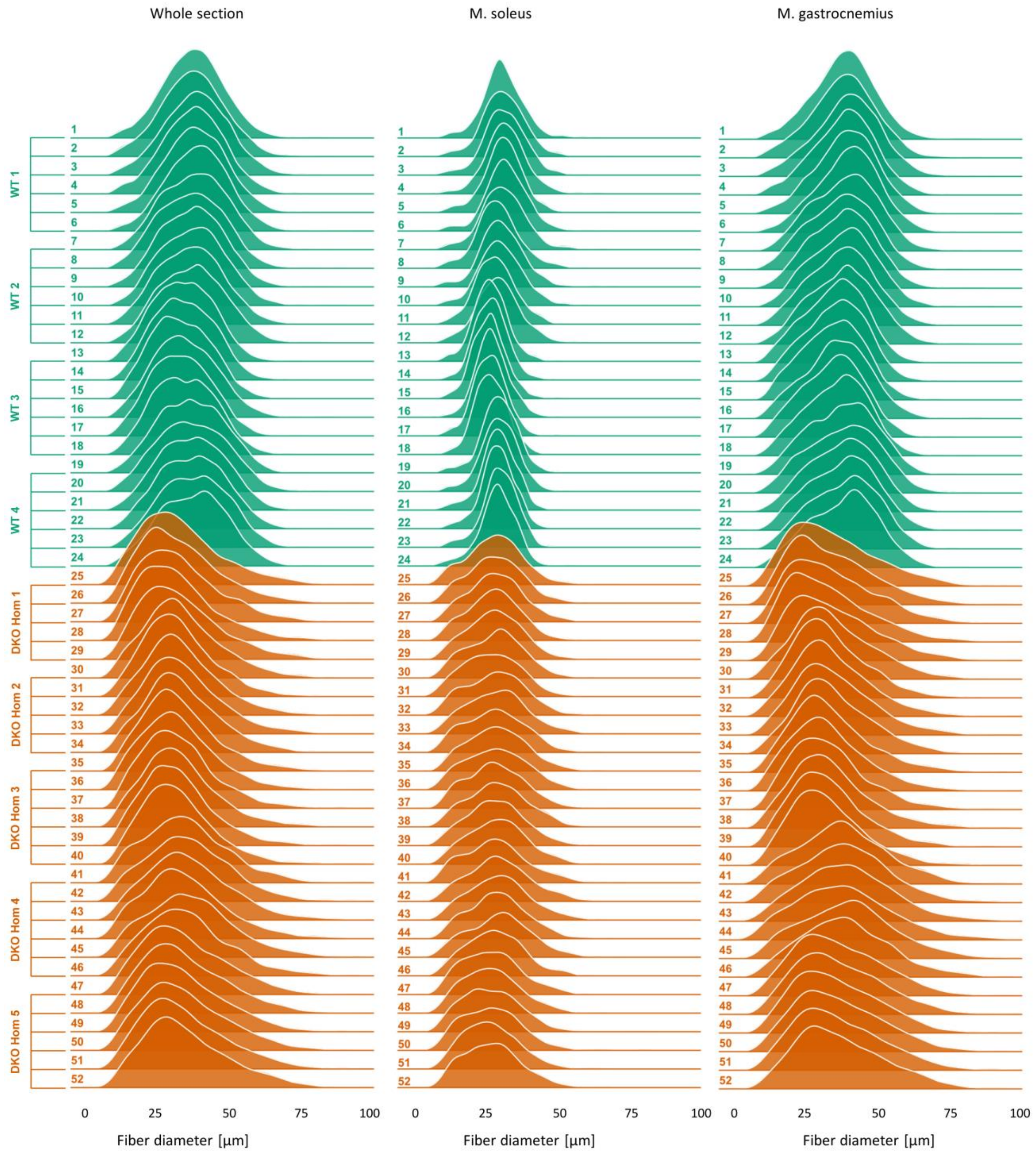

Supp. Fig. 6 | Kernel density estimation (KDE) plots of the fiber diameter distributions of each WT (green) and DKO Hom (orange) WSI section. The KDEs are visualized separately either for the whole section, the M. soleus only or the M. gastrocnemius only. Additionally, the sections are grouped by the tissue samples, wild-type siblings WT 1 – WT 4 and desmin knock-out animals DKO Hom 1 – DKO Hom 5. The plots visualize an increased variance of fiber diameter distribution and a decrease of mean fiber diameter in desmin knock-out skeletal muscle.
