# Supplementary material for: Deep learning-based image analysis in muscle histopathology using photo-realistic synthetic data
Source: Commun Med (Lond). 2025 Mar 6;5:64. doi: 10.1038/s43856-025-00777-y (PMC11885816; doi:10.1038/s43856-025-00777-y)
Supplement: Supplementary file 2 — Description of Additional Supplementary Files [file 43856_2025_777_MOESM2_ESM.pdf]

Description of Additional Supplementary Files

File name: Supplementary Data 1-2

File description: source data for the plots in Fig. 3

File name: Supplementary Data 3-8

File description: the source data for the plots in Fig. 5

File name: Supplementary Data 9-10

File description: the source data for the plots in Fig. 6
