# Supplementary material for: Deep learning-based image analysis in muscle histopathology using photo-realistic synthetic data
Source: Commun Med (Lond). 2025 Mar 6;5:64. doi: 10.1038/s43856-025-00777-y (PMC11885816; doi:10.1038/s43856-025-00777-y)
Supplement: Supplementary file 13 — Reporting Summary [file 43856_2025_777_MOESM13_ESM.pdf]

nature portfolio

Corresponding author(s): M.Sc. Leonid Mill (lmill@mira.vision); Prof. Dr. Anika Grüneboom (anika.grueneboom@isas.de)

Last updated by author(s): Feb 6, 2024

# Reporting Summary

Nature Portfolio wishes to improve the reproducibility of the work that we publish. This form provides structure for consistency and transparency in reporting. For further information on Nature Portfolio policies, see our Editorial Policies and the Editorial Policy Checklist.

## Statistics

For all statistical analyses, confirm that the following items are present in the figure legend, table legend, main text, or Methods section.

| n/a | Confirmed | |
|---|---|---|
| ☐ | ☒ | The exact sample size (*n*) for each experimental group/condition, given as a discrete number and unit of measurement |
| ☐ | ☒ | A statement on whether measurements were taken from distinct samples or whether the same sample was measured repeatedly |
| ☐ | ☒ | The statistical test(s) used AND whether they are one- or two-sided<br>*Only common tests should be described solely by name; describe more complex techniques in the Methods section.* |
| ☒ | ☐ | A description of all covariates tested |
| ☐ | ☒ | A description of any assumptions or corrections, such as tests of normality and adjustment for multiple comparisons |
| ☐ | ☒ | A full description of the statistical parameters including central tendency (e.g. means) or other basic estimates (e.g. regression coefficient) AND variation (e.g. standard deviation) or associated estimates of uncertainty (e.g. confidence intervals) |
| ☐ | ☒ | For null hypothesis testing, the test statistic (e.g. *F*, *t*, *r*) with confidence intervals, effect sizes, degrees of freedom and *P* value noted<br>*Give P values as exact values whenever suitable.* |
| ☒ | ☐ | For Bayesian analysis, information on the choice of priors and Markov chain Monte Carlo settings |
| ☒ | ☐ | For hierarchical and complex designs, identification of the appropriate level for tests and full reporting of outcomes |
| ☒ | ☐ | Estimates of effect sizes (e.g. Cohen's *d*, Pearson's *r*), indicating how they were calculated |

*Our web collection on statistics for biologists contains articles on many of the points above.*

## Software and code

Policy information about availability of computer code

| | |
|---|---|
| Data collection | Zeiss Axio Lab.A1 laboratory microscope using the cellSens Entry Software (OLYMPUS, version 1.3).<br>Hamamatsu NanoZoomer S60 (C13210).<br>Individual settings for data acquisitions via the systems listed above are described in detail in the experimental procedures. |
| Data analysis | t-SNE algorithm was applied using the scikit-learn (version 0.24.0) Python machine learning framework.<br>The deep learning networks were implemented and trained using the PyTorch machine learning framework (version 1.10.0).<br>Fiji (version v1.53f51) and QuPath (0.3.0) were used for image visualization and image processing.<br>The statistical test were performed using the SciPy (version 1.4.1.) python scientific computing library.<br>The open-source collaborative web environment Cytomine was used during the expert study for visualization purposes.<br><br>All data analysis strategies and softwares are described prescisely in the experimental procedures. |

For manuscripts utilizing custom algorithms or software that are central to the research but not yet described in published literature, software must be made available to editors and reviewers. We strongly encourage code deposition in a community repository (e.g. GitHub). See the Nature Portfolio guidelines for submitting code & software for further information.

## Data

Policy information about availability of data

All manuscripts must include a data availability statement. This statement should provide the following information, where applicable:

- Accession codes, unique identifiers, or web links for publicly available datasets
- A description of any restrictions on data availability
- For clinical datasets or third party data, please ensure that the statement adheres to our policy

The data that support the findings of this study are available on reasonable request from the corresponding author [L.M., A.G.].

## Human research participants

Policy information about studies involving human research participants and Sex and Gender in Research.

| | |
|---|---|
| Reporting on sex and gender | N / A |
| Population characteristics | N / A |
| Recruitment | N / A |
| Ethics oversight | N / A |

Note that full information on the approval of the study protocol must also be provided in the manuscript.

# Field-specific reporting

Please select the one below that is the best fit for your research. If you are not sure, read the appropriate sections before making your selection.

☒ Life sciences ☐ Behavioural & social sciences ☐ Ecological, evolutionary & environmental sciences

For a reference copy of the document with all sections, see nature.com/documents/nr-reporting-summary-flat.pdf

# Life sciences study design

All studies must disclose on these points even when the disclosure is negative.

| | |
|---|---|
| Sample size | Sample size was determined by statistical power analysis including high signficance levels (p < 0.05). Box-plot diagrams display means and quartiles with whiskers from minimum to maximum. Scatter plots display means and standard deviations. Diverging stacked bar charts were analyzed using paired Student's t-test. |
| Data exclusions | No data were excluded from analysis. |
| Replication | Minimum five independent measurements per experiment were performed and successfully confirmed results. |
| Randomization | Experimental groups were randomly allocated. DKO mice were housed together with WT control groups. |
| Blinding | Expert study and data analysis was performed in a blinded fashion. The results were confirmed by two investigators, who analyzed the blindet data independently. |

# Reporting for specific materials, systems and methods

We require information from authors about some types of materials, experimental systems and methods used in many studies. Here, indicate whether each material, system or method listed is relevant to your study. If you are not sure if a list item applies to your research, read the appropriate section before selecting a response.

## Materials & experimental systems

| n/a | Involved in the study |
|---|---|
| ☒ | ☐ Antibodies |
| ☒ | ☐ Eukaryotic cell lines |
| ☒ | ☐ Palaeontology and archaeology |
| ☐ | ☒ Animals and other organisms |
| ☒ | ☐ Clinical data |
| ☒ | ☐ Dual use research of concern |

## Methods

| n/a | Involved in the study |
|---|---|
| ☒ | ☐ ChIP-seq |
| ☒ | ☐ Flow cytometry |
| ☒ | ☐ MRI-based neuroimaging |

# Animals and other research organisms

Policy information about studies involving animals; ARRIVE guidelines recommended for reporting animal research, and Sex and Gender in Research

| | |
|---|---|
| Laboratory animals | In this study the following mouse lines were used:<br><br>- C57BL/6<br>Strain: C57BL/6J, Source: Charles River, Identifier: 632<br><br>- homozygous desmin-knockout (DKO) mice and wild-type siblings<br>Strain: B6J.129S2/Sv-Destm1Cba/Cscl<br><br>All mice were housed under "specific and opportunistic pathogen-free" (SOPF) condititions at the animal facilities of the University of Erlangen, Germany. |
| Wild animals | This study does not include wild animals. |
| Reporting on sex | Both sexes were included in this study. 15 weeks old male wildtype C57BL/6J and 28-34 weeks old female homozygous desminknockout (DKO) mice and female wild-type siblings were used. |
| Field-collected samples | This study does not include field-collected samples. |
| Ethics oversight | Sample preparation was carried out in compliance with all ethical regulations for organ removals at the University of Erlangen and carried out as approved by the local animal ethic committees of the Regierung von Mittelfranken (reference number 55.2.2-2532-2-1073), and the governmental office for animal care (Landesamt für Natur, Umwelt und Verbraucherschutz North Rhine-Westphalia (LANUV NRW), Recklinghausen, Germany (reference numbers 84-02.04.2014.A262 and 84-02.05.40.14.057)). |

Note that full information on the approval of the study protocol must also be provided in the manuscript.
